# Photocatalysis and perovskite oxide-based materials: a remedy for a clean and sustainable future

**DOI:** 10.1039/d1ra08185c

**Published:** 2022-03-02

**Authors:** Muneeb Irshad, Quar tul Ain, Muhammad Zaman, Muhammad Zeeshan Aslam, Naila Kousar, Muhammad Asim, Muhammad Rafique, Khurram Siraj, Asif Nadeem Tabish, Muhammad Usman, Masood ul Hassan Farooq, Mohammed Ali Assiri, Muhammad Imran

**Affiliations:** Department of Physics, University of Engineering and Technology Lahore 54890 Pakistan muneebirshad@gmail.com muneeb_irshad@uet.edu.pk; Department of Physics, University of Sahiwal Sahiwal Pakistan; Department of Chemical Engineering, University of Engineering and Technology, New Campus Lahore Pakistan; Department of Mechanical Engineering, University of Engineering and Technology Lahore 54890 Pakistan; Department of Basic Sciences, University of Engineering and Technology, New Campus Lahore Pakistan; Department of Chemistry, Faculty of Science, Research Center for Advanced Materials Science (RCAMS), King Khalid University P. O. Box 9004 Abha 61413 Saudia Arabia

## Abstract

The massive use of non-renewable energy resources by humankind to fulfill their energy demands is causing severe environmental issues. Photocatalysis is considered one of the potential solutions for a clean and sustainable future because of its cleanliness, inexhaustibility, efficiency, and cost-effectiveness. Significant efforts have been made to design highly proficient photocatalyst materials for various applications such as water pollutant degradation, water splitting, CO_2_ reduction, and nitrogen fixation. Perovskite photocatalyst materials are gained special attention due to their exceptional properties because of their flexibility in chemical composition, structure, bandgap, oxidation states, and valence states. The current review is focused on perovskite materials and their applications in photocatalysis. Special attention has been given to the structural, stoichiometric, and compositional flexibility of perovskite photocatalyst materials. The photocatalytic activity of perovskite materials in different photocatalysis applications is also discussed. Various mechanisms involved in photocatalysis application from wastewater treatment to hydrogen production are also provided. The key objective of this review is to encapsulate the role of perovskite materials in photocatalysis along with their fundamental properties to provide valuable insight for addressing future environmental challenges.

## Abstract 2

Photocatalytic reaction for CO_2_ reduction in presence of co-catalyst.

## Graphical abstract

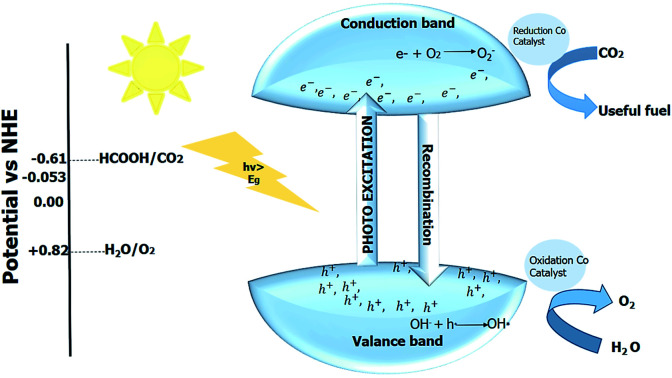

## Introduction

Rapid industrialization and population growth resulted in increased energy resources utilization and release of harmful pollutants. The increased human activities are depleting the energy resources and polluting our ecosystem. The toxic gasses in the air are causing respiratory diseases, and the direct disposal of industrial and organic waste into the water reservoirs is causing water-borne diseases. Nearly 663 million people in the world have no access to clean water. The amount of CO_2_ in the atmosphere is also increasing due to rapid industrialization. The increased concentration of CO_2_ is causing is global warming and is the leading cause of acid rain, which is dangerous for all living things on the earth. The current decade 2021–2030, is the decade of ecosystem system restoration. Therefore, it is essential to pay attention to clean ways to tackle environmental issues. The new global challenge is the achievement of ecological sustainability. The research focus of this era is oriented toward the way to achieve the eco-friendly system.^1–6^

The blissful gift of Mother Nature, the Sun is an ultimate renewable energy resource that irradiates 3.85 yotta joule (YJ) of energy yearly on the earth's surface. Sunlight is one of the best routes to deal with environmental issues owing to its cleanness and abundant availability. Sunlight can be utilized as an energy medium by photovoltaic, photoelectrochemical catalysis, or photocatalysis in daily activities.^7,8^

Photocatalysis is defined as the science of employing a catalyst that uses light to speed up the chemical reaction. In photocatalysis, the photocatalyst material is used, and the energy of the light acts as a source to generate electron–hole pair. The photogenerated electron–hole pair then initiates the redox reaction at the surface of the photocatalyst. This redox reaction can be utilized to degrade water pollutants and convert abundant earth elements (H_2_O, CO_2,_ and N_2_) into fuel (pure H_2_, or organic fuel like CH_4_, CH_3_OH, and NH_3_). The conversion of water into the fuel (H_2_) by using light and photocatalyst material is known as photocatalytic water splitting. The conversion of CO_2_ into the hydrocarbons is known as CO_2_ reduction, and conversion of nitrogen into NH_3_ is called nitrogen fixation. All these application deals with the viable way to clean the environment. The main challenge while dealing with photocatalysis is the region of operation of the photocatalyst in the solar spectrum. Most photocatalysts operate under UV radiations, which is only 5% of the sunlight reaching earth. Therefore, suitable photocatalysts are required to respond to the extensive range of the solar spectrum.^9–18^

## History of photocatalysis

The word catalysis was first used in 1836 by the Jöns Jakob Berzelius, derived from the Greek word “kata”, meaning down or loosen. Catalysis is the process of fasting or accelerating the reaction in the presence of the catalyst. The catalyst itself doesn't participate in the reaction but increases the reaction rate. The term photocatalysis first appeared in 1911, when Eibner studied the effect of light irradiation on transition metal oxide (ZnO) for the decolorization of Prussian blue. Photocatalysis term also appeared simultaneously in the title of the article investigating the degradation of the oxalic acid in the presence of the uranyl salts upon light irradiation. In 1921 Edward Charles Cyril Baly studied the production of formaldehyde using colloidal uranium salts and ferric hydroxides as a catalyst. Soon after, in 1924, the Baur and the Perret at the ETH of Zurich reported the effect of light irradiation on the ZnO suspension and observed that ZnO suspension enhanced reduction of Ag^+^ salts to Ag^o^ upon irradiation. The mobility for the TiO_2_ as a photosensitizer for the dyes was first studied by Doodeve and Kitchener in 1938. This work reported that TiO_2_ upon light irradiation decolorized the dyes, and TiO_2_ remains unchanged during the process.^19–22^

Many researchers from 1940 to 1972 investigated different parameters of the photocatalyst. Photocatalysis gained significant attention when electrochemical photolysis of the water was done by using titanium dioxide under UV irradiation. The oil crisis in 1973 also changed the social and economic status of the western world. The first time, the shortage of fossil fuels became a significant issue, which led to an unprecedented increase in the efforts for alternative energy resources, including photocatalysis. The application of photocatalysis is not limited to energy fuels but can also clean the environment. Much attention has been given to photocatalysis during the last two decades due to its wide range of applications.^21,23^

Photocatalysis has its application in the pollutant degradation and production of fuel. Material can be an excellent photocatalyst if it is responsive to light, especially (UV or visible), biologically and chemically inert, has low cost, and exhibits adsorption and absorption capacity. Normally the metal oxide semiconductors are used in the application of the photocatalyst. Perovskite materials, due to their unique optical properties, also gained importance in the application of photocatalyst during the last decade.^24,25^

## Perovskite oxide based materials

**Fig. 1:**
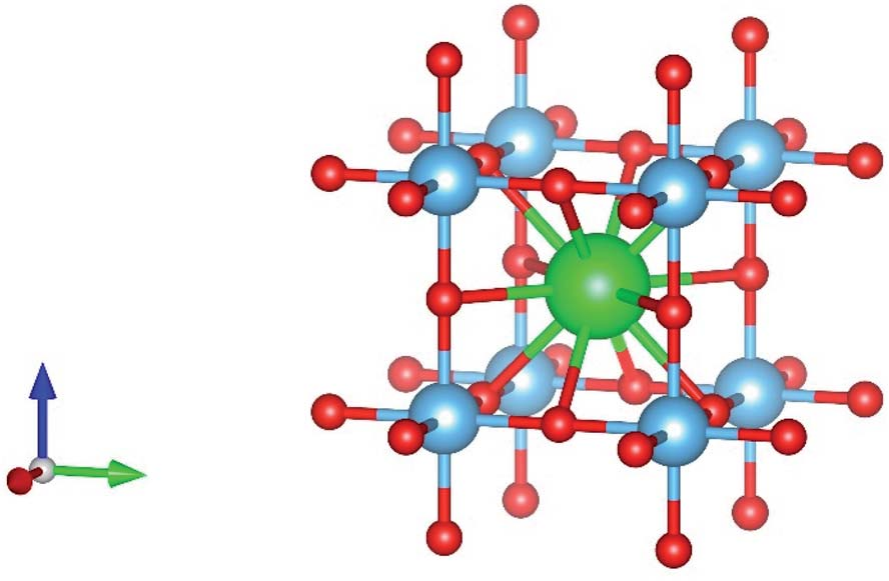
Perovskite structure.

Perovskite material was first discovered in 1839 in the Ural Mountains by the German scientist Gustave Rose. The mineral was named perovskite after Russian mineralogist Count Lev Aleksevish von Perovski.^26–28^ The first discovered perovskite material was CaTiO_3_.^29^ The basic chemical formula of the single perovskite is ABX_3_, where A and B are cations while X is an anion. Normally, A is alkaline earth metals or lanthanides, B is the transition metal, and X is oxygen or any halide. The perovskite is known as perovskite oxide when the anion is oxygen and is known as perovskite halide when the anion is a halide. In single perovskite, the coordination number of A site is 6, the coordination number of B site is 12, and the coordination number of O anion is 6. In single perovskite, the perfect structure of the BO_6_ octahedral connection results in the cubic lattice.^30–33^ The structure of single perovskite is shown in Fig. 1.

The tolerance factor can indicate the distortion and the crystallographic structure of the perovskite material. The tolerance factor of the single perovskites ions was defined by V. M Goldschmidt in 1926. The tolerance factor of the perovskite structure tells the stability of the perovskite structure and the compatibility of the ions in the crystal structure.^34–39^ The tolerance factor is a dimensionless quantity given by eqn (1).^38,40^1
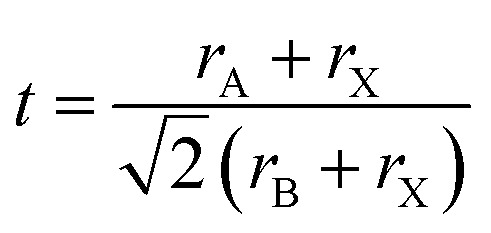


In eqn (1), *r*_A_ and *r*_B_ are the ionic radii of the electropositive ions, while *r*_X_ is the radius of the oxygen or halide ions.^41^ The perovskite structure can be divided into octahedral hexagonal, tetragonal, and ideal cubic structures on the basis of the tolerance factor. When the tolerance factor is between 0.9 and 1, the structure is the ideal cubic structure with the length of the unit cell (*a*) as:^42^2
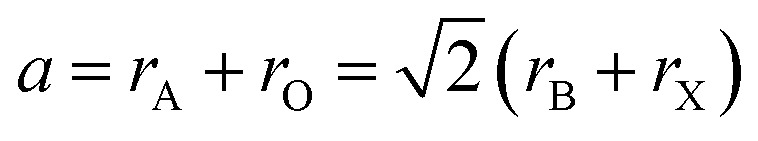


In non-ideal cases, the tolerance factor deviates from 1, which shows the mismatch in the A–O and B–O bond lengths. The corundum structure (α-Al_2_O_3_) and its derivative are considered when the tolerance factor is less than 0.75.^43^ The stable bixbite polymorph (α-Mn_2_O_3_) is favored when the factor decreases. If the tolerance factor is greater than 1, the structure of perovskite is hexagonal close-packed (HCP), and [BO_6_]-octahedra share faces with the hexagonal *c*-axis.^30,44^

The sum of the charges of cations and anion of the perovskite structure should equal one for perovskite material to be electrically neutral. Different cations having different ionic radii and valences can be doped in the structure of perovskite material by employing the method of partial substitution at A and B-site. Deficiency at A and B-site cations and excess or deficiency of oxygen anions can change the composition of ions and the non-stoichiometry phenomenon.^45,46^

**Fig. 2:**
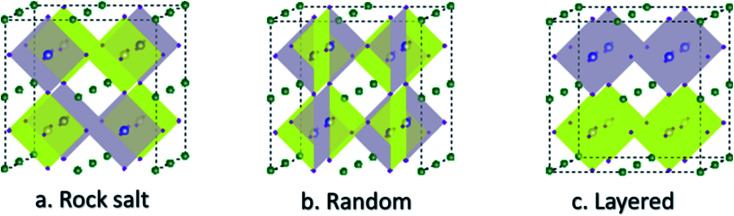
Double perovskite structures.

Double perovskite structures were used at a significant scale in the 1980s. The double perovskite exhibits structure that is twice the single perovskite. The coordination number of the A and B cation is the same as that of the single perovskite. Double perovskites are generally of two types depending upon the types of the cations A′A′′B_2_O_6_ (double A-site) or A_2_B′B′′O_6_ (double B-site).^47,48^ The double perovskite strain energy depends on the charge difference between the two types of B cations (B and B′). There are three ways to arrange the B-type cation based on the charge difference between B and B′. The arrangement is random if the charge difference is one (Δ*Q* = 1).^49^ The most common arrangement of the double perovskite is the rock-salt arrangement, also called elpasolite structure, in which the cations change in all three dimensions, and it dominates when the charge difference between the B and B′ is greater than 2 (Δ*Q* > 2). Due to the difference in the charge size of the B cation in the rock salt order, the crystal symmetries are less than their single perovskite structure. Another arrangement is the layered arrangement of the B and B′, where the cations can change in only one dimension (Fig. 2).^50–54^

The perovskite structures are preferred due to their structural and compositional flexibility.

### Structural flexibility

The ideal single perovskite structure is with the high symmetry of *Pm*3̄*m*. It comprises a highly flexible network built up from chains of corner-sharing [BO_6_] octahedra with A cations occupying the resulting holes with cubic octahedral symmetry. The symmetry of the structure can be transformed into the tetragonal, hexagonal, octahedral, monoclinic, triclinic, and rhombohedral structures by changing the size of cations or anions in the structure. Double perovskites generally have a double B site. Double perovskites are modified single perovskite; the high symmetry ideal single perovskite with the space group of *Pm*3̄*m* is reduced to *Fm*3*m* in double perovskite. The double perovskite structure with double B site has two elements in the corner linked and has alternatively arranged BO_6_ and B′O_6_ octahedra double A site.^55,56^

The tolerance factor of both single and double perovskite changes by altering the size of cations or anions in the perovskite. The resulting lattice distortion affects the dielectric, electronic, magnetic, and optoelectronic properties. The lattice distortion also influences the movement and excitation of the photo-generated charge carrier under the influence of light.^27,57–59^ The sintering temperature also affects the structure of the perovskite material. The structure generally becomes ideal cubic at high temperatures, and as the temperature goes down, the octahedral rotation in the perovskite changes to lower symmetry.^60,61^

Octahedral tilting also has an indispensable role in the structural flexibility of single and double perovskite structures. Octahedral tilting is the rotation along with the orthogonal symmetry of the BO_6_; Glazier explained the octahedral tilting in 1972. Currently, there are twenty-three different perovskite structures available depending on the octahedral tilt.^62,63^

The ferroelectric properties of the perovskite structure are also dependent on the octahedral tilting. Octahedral tilting breaks the centro-symmetry, which affects the ferroelectricity. The bandgap of the perovskite can also be changed by changing the octahedral tilting. Calculation of the electronic structure of the oxide perovskites on the tilted phase depicts that bandgap due to the different tilt can change up to the 0.2 eV and is even more significant in the case of halide perovskites.^30,64,65^

The large distortion in the perovskite lattice can break the structure and result in low dimension perovskite formation (1D, 2D). The distortion in the crystal structure affects the BO_6_ octahedral, breaks the B–O bond, and forms a 1D/2D perovskite derivative. In 1D wires with linear or zigzag configuration, the BO_6_ octahedral network can be edge-sharing, face-sharing, or corner-sharing. While in the case of a 2D stacked-layer, the BO_6_ octahedra are edge-sharing. Low dimension perovskites are helpful in the application of photocatalysis due to their high surface to bulk ratio.^65–67^

#### Stoichiometric and compositional flexibility

The perovskite structure exhibits a high degree of stoichiometric and compositional flexibility. Theoretically, 346 different kinds of ABO_3_, 264 are experimentally investigated. The ABO_3_ perovskite structure is divided into five groups depending upon the A and B site charges that are A^1+^B^5+^O_3_, A^2+^B^4+^O_3_, A^3+^B^3+^O_3_, A^4+^B^2+^O_3_, and A^5+^B^1+^O_3_. Theoretically, there are 10^5^ possible material double perovskite, out of which 10^3^ double perovskites are experimentally investigated. For material to be double perovskite, the charge balance of all the charges present in the structure should be equal to 12. Double perovskite allows the elements with high valence shells to accommodate in structure, thus expanding the compositional flexibility.^49,68^

Perovskites' compositional flexibility is often represented by their ease of alloying on A and B sites. Normally, the mixing element in semiconductors is isovalent, while in perovskite, the mixing element is non-isovalent. The simplicity of cation mixing in perovskite materials makes it easy to alter its chemical and physical characteristics. The double perovskite structure also shows interesting properties like half-metallicity, high-temperature ferromagnetism, and many magnetic interactions. The infrastructure of the complicated oxide unit cell depends upon the electronic configuration and the atoms located at the A and B-site. The B-site atoms are considered to be more critical than A-sited cations. The size of the A^2+^ cation is larger compared to the A^3+^ cation. As the A^3+^ cation has a smaller size, it limits the size of B-site cations. More than one thousand double perovskite materials are reported in the literature, all of which are prepared at ambient pressure. Some new materials were synthesized at high pressure. More than 720 compounds are reported as the divalent A-site compounds and 200 as trivalent A-site.^30,69–71^

The structural network of BO_6_ octahedra can be maintained in the presence of vacancies at A, B, and O sites because of the perovskite's excellent structural and compositional flexibility. Applications of the perovskite materials are broad due to these vacancies, and therefore, they carry applications in photocatalysis, photovoltaics, supercapacitors, batteries and fuel cells, *etc.*^30^

#### Stability of perovskite oxide materials

The challenge to make use of perovskite photocatalyst materials come from two significant restraints: (1) chemical instability in polar solvents and (2) vulnerability of the surface to transform with chemical species present in solution. Recent progress in this field improved these issues and illustrated different courses of actions to make use of perovskite materials in photocatalytic processes.^72^ There are two main stability issues; (1) intrinsic stability–stability issues, and (2) extrinsic stability–stability issues of perovskite. Intrinsic stability involves structural stability, electronic band structure, and thermodynamic phase stability. In contrast, extrinsic stability encompasses interaction with water molecules and pathways of degradation, thermochemical stability, light induced ion redistribution, light stability, photochemical degradation, and oxidation/photo-oxidation of charge-transporting layers and perovskites.^73^

**Fig. 3:**
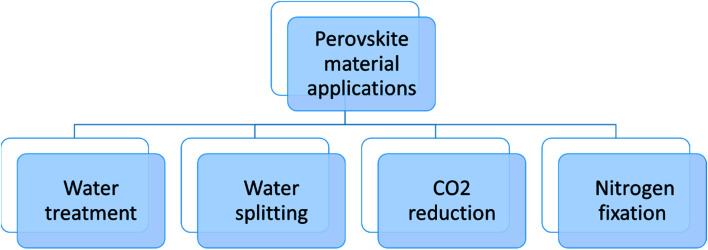
Applications of perovskite materials.

#### Perovskite material and photocatalysis

Perovskite materials, due to their structural, compositional, and stoichiometric flexibility, are considered promising materials for photocatalysis (Fig. 3). Perovskites-based photocatalysts operate in UV, IR, and visible regions. There are three different sites of alteration in perovskite material which makes tuning of the bandgap in perovskite material easy. In this review, the applications of the perovskite material in photocatalysis are discussed in detail.^74,75^

#### General reaction mechanism of photocatalysis

The light interacts with the surface of the nano photocatalyst, which generates the electron (e^−^) and hole (h^+^) pair. The photogenerated electron–hole pair then initiates the redox reaction at the photocatalyst's surface. There are three steps involved in the mechanism of photocatalysis. The first one is photo-excitation, provided that the energy of the light source should be high enough to overcome the bandgap. The second step is trapping the electron–hole pairs to increase the recombination time. The recombination of photoinduced charges produces heat, which decreases the efficiency. The third and final step is the degradation of the pollutant by the reactive species produced due to a redox reaction or the production of the fuel.^76–79^ The general reaction mechanism for all the reactions is given by;

1st step: photoexcitation of photocatalyst

3 Photo catalyst + *hv* → e^−^ + h^+^

2nd step: trapping of electron and hole:4
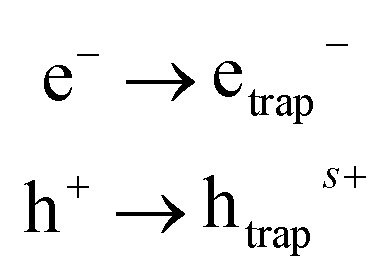

5 Oragnics (pollutant) + radicals → degarded pollutant

The final step varies for each application of photocatalysis and governing equations:

For water splitting:

6 H^+^ + e^−^ → H_2_

For CO_2_ reduction:

7 H^+^ + e^−^ + CO_2_ → fuel (H_2_ + CH_4_)

For nitrogen fixation:

8 6H^+^ + 6e^−^ + N_2_ → NH_3_

**Fig. 4:**
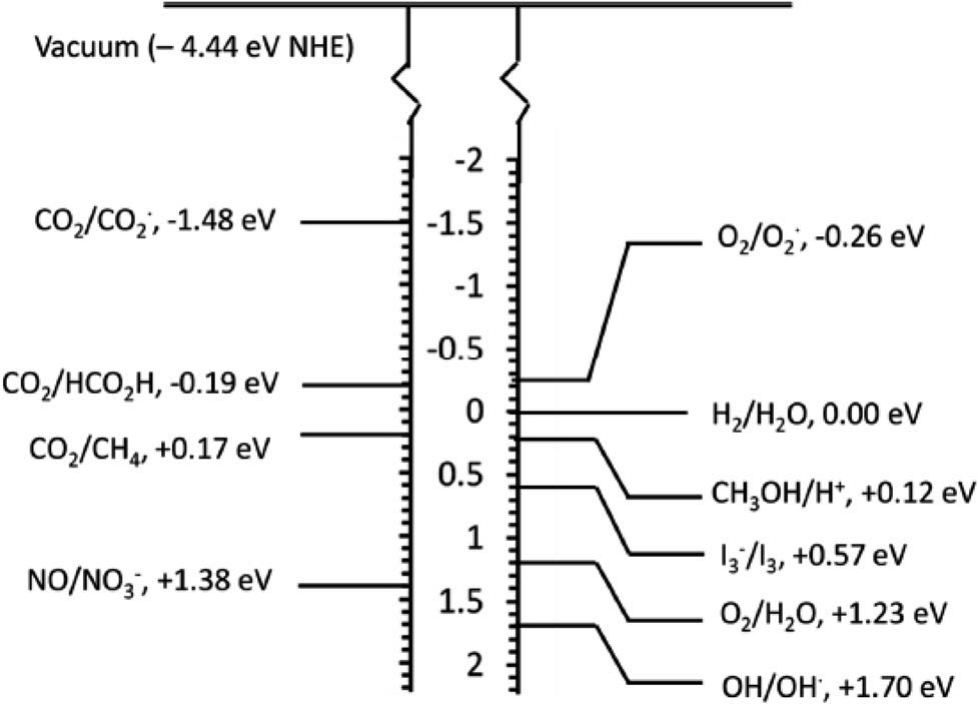
Redox potential required for the various photocatalytic applications.^10^

The photogenerated electron–hole pair can be used in various applications depending upon the redox potential for the applications, as depicted in Fig. 4.

The selection of the photocatalyst material for the particular application is crucial and an important step to achieve better results.^80^ The semiconductor material is selected by balancing the band edge potential of the semiconductor material with the reaction potential. The main hurdles in photocatalysis are the less recombination time of the photoinduced electron–hole pair and absorption wavelength corresponding to the photocatalyst material. Most materials are UV activated, and in the solar spectrum, the UV region is only 5%; therefore, for enhanced photocatalytic activity, the material bandgap should be responsive to visible and NIR radiations along with the high recombination time of electron–hole pair.^81,82^

In the perovskite structure, there are three sites of the alteration (A site, B site, and O site). The doping at these sites can enhance light absorbance in the visible and NIR regions by bandgap alteration. The recombination time can also be addressed by employing the different strategies that includes tuning of bandgap and repression of electron–hole recombination. To solve both light absorption and recombination time problems, a new innovative solution known as defect engineering has been proposed.^8,83–85^ The strategies for tuning the bandgap are;

(i) Bandgap engineering

(ii) Repression of electron–hole pair recombination

(iii) Defect engineering

The bandgap of the various perovskite materials is given in Table 1.

**Table 1:** Bandgap of some perovskite materials

Material	Bandgap (eV)	Ref.
SrTiO_3_	3.20	86
NaTaO_3_	4.0	87
CaTiO_3_	3.62	88
BiFeO_3_	2.40	89
LaFeO_3_	2.00	90
NaNbO_3_	3.48	91
LaCoO_3_	2.1	92
Bi_2_WO_6_	2.70	93
La_2_Ti_2_O_7_	3.28	94

##### Bandgap engineering

**Fig. 5:**
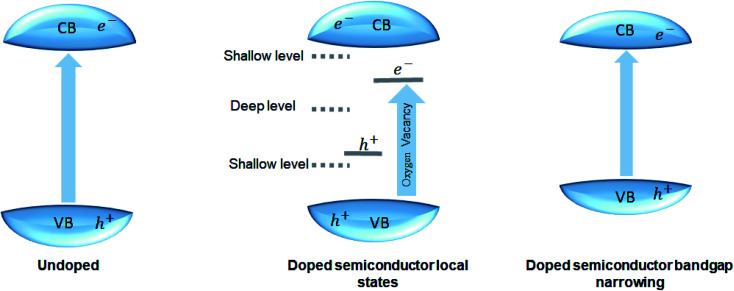
Effect of doping on the bandgap.

Most perovskite materials respond to UV light due to their wide-bandgap. The conduction band of perovskite materials contains d-orbital, slightly above or equal to zero eV. The valence band has the 2p orbital, due to which energy of the valence band in perovskite material is generally greater than 3 eV. The perovskite bandgap provides enough redox potential to execute different photocatalytic reactions, like H_2_ generation, CO_2_ reduction, contaminants degradation, and nitrogen fixation. The donor and acceptor impurities in the perovskite crystal structure can adjust the bandgap. The donor and acceptor impurities in a perovskite can tune the material's bandgap. The doping at the cations or anions at different sites of perovskite structure enhances the photocatalytic activity by introducing the intra-band energy levels; the shallow and deep intraband energy levels trap the photogenerated electron–hole pair. Doping also reduces the bandgap of the perovskite material (Fig. 5).^30,95^

In perovskite material, doping of the transition metals (d-electrons rich cations) led to the production of donor impurities in the perovskite structure. The high atomic energy of the transition metal ensures better electron mobility without changing conduction band minima of perovskite host material.^96^

The doping at the different sites of the perovskite structure changes the absorption peak of the material, thus increasing the light absorption. Doping also tailors the material's bandgap and increases the efficiency of the photocatalyst material. Incorporating a new transition energy level in the perovskite structure improves the higher wavelength absorption without changing the intrinsic absorption properties of the material. Hybridization of orbital in perovskite structure and formation heteroatom due to the doing changes the absorption spectra of material. The hybridization decreases the bandgap and therefore increases the absorption of light. Thus, the doping changes the bandgap and increases the recombination time.^83,97–99^

The bandgap of pure SrTiO_3_ is 3.25 eV, and doping with noble metals like Ru, Rh, Au, *etc.*, introduces the intra-band local states, making the doped SrTiO_3_ a suitable photocatalyst.^100^ The Ru doping introduces the intra-band energy state between conduction band (Ti 3d) and valence band (O 2p, Sr 4p). The ionic radius of Ru is greater than Sr and nearly equals Ti; therefore, the Ru substitutes Ti atom in SrTiO_3_. Ru doping at the B site of the SrTiO_3_ introduces the local states and increases the photocatalytic performance. The doping of noble metals in SrTiO_3_ makes it a Vis-IR responsive photocatalyst.^86^

Similarly, doping the bismuth (Bi) or copper (Cu) at A site of SrTiO_3_ introduces the intra-band energy level and reduces the bandgap. The change in the absorption shift was also reported due to the change in the bandgap with doping.^101^ It is reported that doping of the sulfur at the O site of the ABO_3_ introduces the intraband energy level at 1.23 eV. Researchers also noted that doping the nitrogen at the O site of the perovskite material increases photocatalytic activity.^102,103^

The amount and the quantity of the dopant should also be optimized to impede the recombination of the photogenerated charge carrier. Synthesis methods for preparing the perovskite material also affect the bandgap, recombination of charges, and migration properties of photocatalyst.^104,105^

##### Repression of the photogenerated electron–hole pair recombination

The movement of photoinduced charge carriers at the photocatalyst's surface initiates the redox reaction at the surface of the photocatalyst. The recombination of charge carriers can occur either at the surface or in bulk. The recombination reduces efficiency, so it must be repressed.^106–108^

The crucial step in photocatalysis is selecting the material, which depends upon the absorption coefficient of the photocatalyst material. The absorption coefficient of photocatalyst depends upon the bandgap and photon energy.^109–111^ The incident light generates the electron–hole pair below the catalyst surface, known as the absorption length. The development of the space charge region takes place at the phase of the solid–liquid interface, and if the absorption extent exists in the space charge region, the photoinduced electron–hole pair can be easily separated. However, in the case of the bulk material, the recombination rate will be high.^112,113^ After separating the photo-induced charges, the next step is the migration of the charge carriers. The migration mainly depends on the material's symmetry and diffusion length. Diffusion length is the distance that photogenerated charges travel without recombining or scattering. Diffusion length is the governing factor for controlling the movement of the charges in the absence of the potential gradient. Separation of the photogenerated charges beyond the charge separation region is feasible with materials whose diffusion length is longer. The diffusion length is multiple of the lifetime of the charge carrier and diffusion constant, and it should be of high value to generate the maximum photogenerated charge carriers.^114–116^ The limiting factor for the range of charge space region is the particle size. The width of the charge space region should not surpass the particle's radius, and therefore the use of nano-size crystals improves the photocatalytic activity.^108,117,118^

The perfection in the crystal structures affects the lifetime of the photogenerated charge carrier. The potential gradient also affects the photocatalytic activity because it efficiently separates the photo-induced charges through the support interfaces between the semiconductor–semiconductor, semiconductor–metal, and semiconductor-conductor surfaces, forming the heterojunctions. Therefore, it is necessary to introduce the potential gradient for attaining better efficiency of photocatalytic material. Furthermore, combining the two semiconductors with different bandgaps represses the electron–hole pair recombination. Co-catalyst can also enhance efficiency by introducing active sites and better charge transfer.^79,119–126^

##### Charge transfer mechanism

**Fig. 6:**
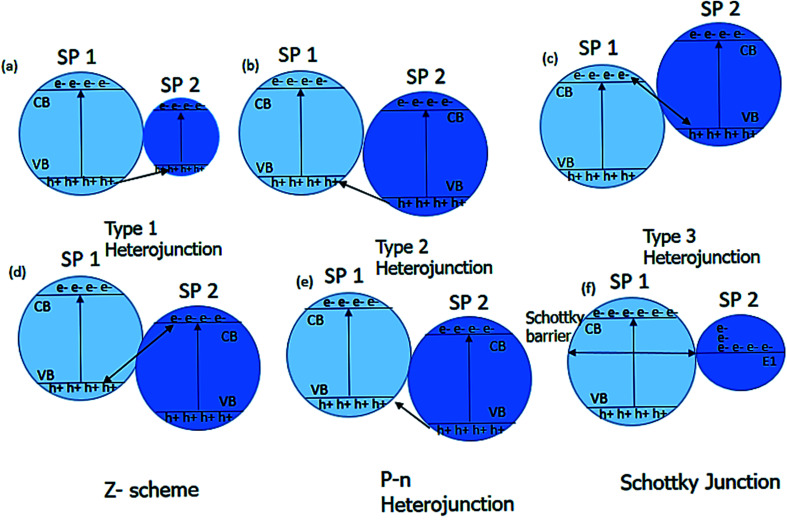
Charge transfer mechanisms (a–f) in two coupled semiconductors.

Charge transfer mechanisms are of different types, depending on the photocatalyst system. The coupling of two photocatalyst semiconductor materials gives the heterojunction, further categorized into three types. The heterojunction type formed by the semiconductor materials depends on their bandgap.^119,127^ The three types of heterojunctions are type I, type II, and type III. The combination of the semiconductor materials forms the Z-scheme, p–n junction, and the Schottky junction based on the type of the semiconductor used (Fig. 6).^121,128,129^

Type I, a heterojunction known as straddling gap heterojunction, involves one semiconductor photocatalyst (SP I) with a highly negative conduction band and highly positive valence band while the bands of the other photocatalyst semiconductor (SP II) lie within it. The drawback of type I heterojunction is that the charges accumulate on the semiconductor with a lower bandgap which is not favorable.^127,130^

In type II, the conduction and valence band of the one semiconductor SPI lies above the conduction and valence band of the SPII. The charge separation of the photogenerated electron–hole pair is efficient in type II because of the electron's downward movement from the conduction band of SPII and an upward movement of the holes from SPI's valence band.^131,132^

In type III heterojunction, the valence and conduction bands difference is more than type II. The photogenerated electrons of the SPI unite with the photogenerated hole of SPII. The SSPII electron and the SPI hole innate the redox reaction in the semiconductor photocatalyst.^120,130^

The understanding of the Z-scheme is necessary to understand the heterojunction. In Z-scheme, two semiconductor photocatalyst materials are linked in a way that they form the letter Z. The electrons from the conduction band of the SPII trans into the valence band of the SPI. SPI acts as a reduction site, and SPII serves as an oxidation site. Due to the redox potential between the two semiconductors, the inbuilt electric field allows efficient charge separation. The direct Z-scheme involves the contact at the interface of two semiconductor materials. In contrast, the indirect Z-scheme involves mediators that allow the charge transfer between the two semiconductor materials. The mediator can be solid or liquid. The iron-based mediators are more commonly used.^133–135^

**Fig. 7:**
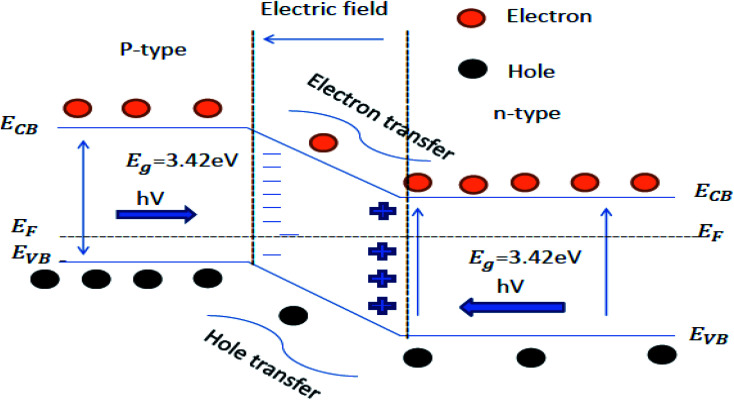
Schematic energy band structure and electron–hole pair separation in the p–n heterojunction.^126^

The heterojunction can also be formed by combining the p and n-type of the semiconductor materials. A free-electron from n-type semi-conductor material migrates toward the p-type semiconductor material upon contact. The migration of free electrons results in the formation of oppositely charged interfaces. This migration also strengthens the inbuilt electric field at the contact of the p–n junction. The built-in electric field facilitates the migration of the photogenerated charge carrier after light irradiation, which increases the photocatalytic performance (Fig. 7).^136,137^ Examples of semiconductor–semiconductor photocatalyst are SnO_2_–TiO_2_, CdS–TiO_2_, Bi_2_O_3_–Bi_2_WO_6_ and Bi_2_WO_6_–TiO_2_, *etc.*^138^

**Fig. 8:**
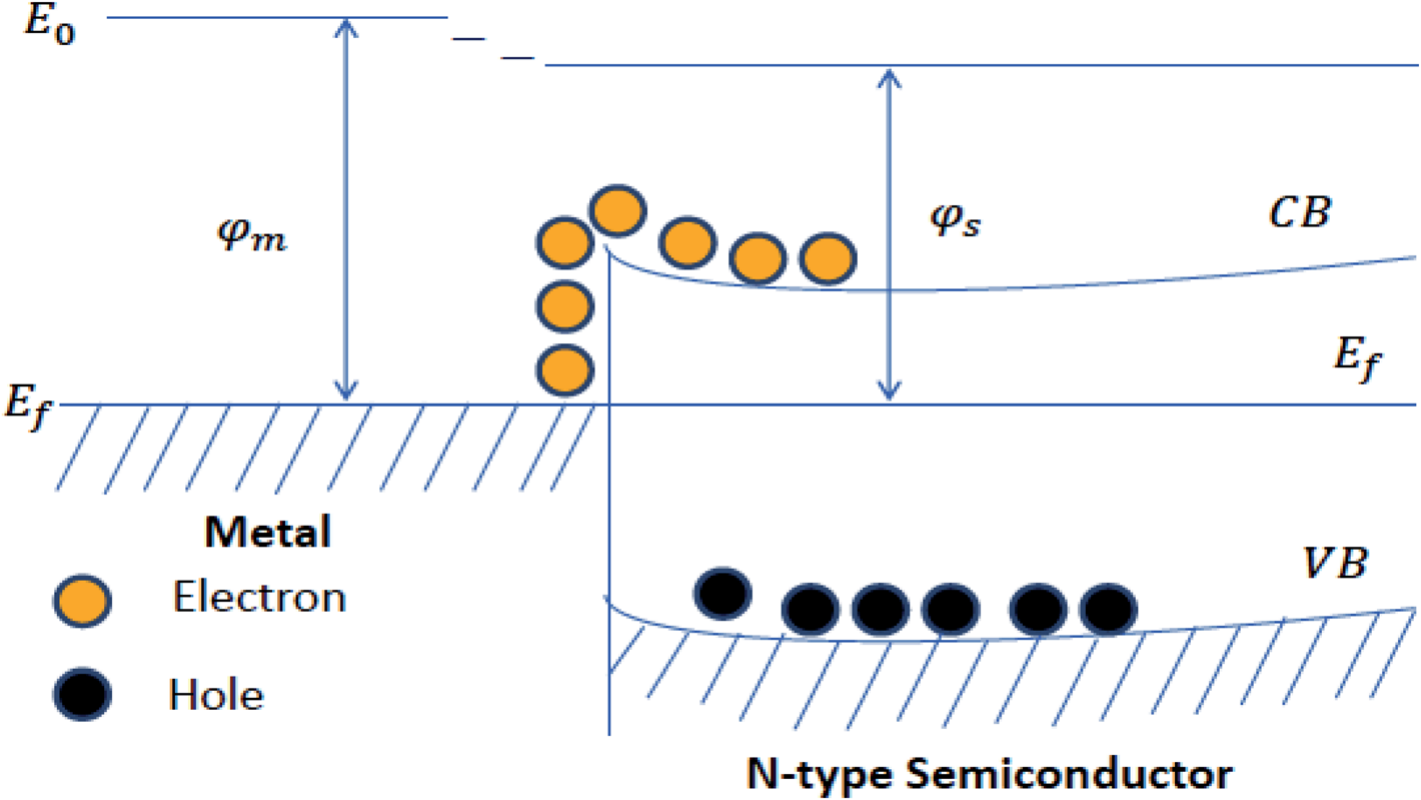
Schematic of the Schottky barrier.^126^

The increased charge separation of metal–semiconductor photocatalysts arises from the transfer of the electron across the interface of metal–semiconductor. Metal improves photocatalytic performance because it provides active sites and facilitates the separation and transfer of photoinduced charge carriers. When a metal (having a high work function) couples with semiconductor, the electrons flow from the semiconductor to the metal until their Fermi levels get aligned, resulting in upward bending of band edges in semiconductor and accompanied by the band bending, formation of Schottky barrier takes place at the metal–semiconductor interface (Fig. 8). The electron from the conduction band of the semiconductor material migrates to the co-catalyst and causes charge separation, and increases the photocatalytic activity.^139–141^ The prominent examples of metal–semiconductor photocatalysts are Au/TiO_2_, Au/ZnO, Ag/ZnO, Ag/TiO_2_, *etc.*^142^

##### Defect engineering

Defect engineering is a suitable and easy way to enhance the photocatalytic efficiency of perovskite materials. Substituting the different materials can introduce defects in the perovskite materials in the perovskite structure. These defects act as a site to trap the charge carriers and thus enhance the separation of charge carriers and increase the photocatalytic efficiency. These defects can be a vacancy of an extra atom that results in the formation of the line or screw dislocation.^143–145^

In perovskite materials, three different alteration sites allow the introduction of the defects or vacancies. The doping at the A and B site cations introduces the dislocation but does not allow structural changes. The structural changes can introduce vacancies at the A site or O site. The vacancy at the B site is challenging to introduce due to the BO_6_ octahedra. Furthermore, it is thermodynamically unfavorable to introduce the vacancy at the B site due to the high charge value and the small B site cation size.^146,147^

**Fig. 9:**
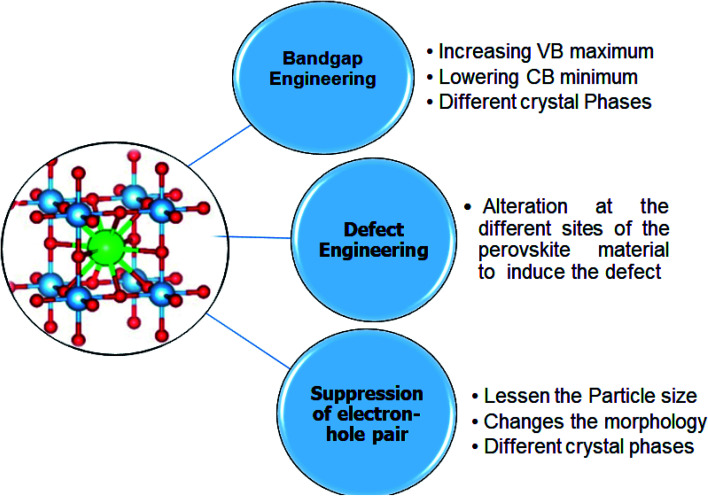
Strategies for tuning bandgap.

The vacancies at the A site can be induced by substituting the A-site cation with a cation of the high or low valence. The subsisted single perovskite oxide formula is: A_1−*x*_A_*x*_BO_3_ and the oxygen-deficient vacancy can be created by doping the sulphur or nitrites. The second approach is doping at the B site, in which the reduction of the B site produces oxygen vacancy. In recent years, many defect-rich perovskite materials with excellent photocatalytic activity have been reported. Fig. 9 represents different strategies for bandgap tunning of perovskite materials.^30,148,149^

The potential of semiconductor photocatalyst mainly depends on the electron injection capacity of the material, which is controlled by the energies of valence (*E*_VB_) and conduction bands (*E*_CB_). Therefore, to investigate the possible applications of semiconductors, it is crucial to have an understanding of the *E*_CB_ and *E*_VB_ band edges. In a semiconductor, the Fermi level (*E*_F_) determines the electrochemical potential of electrons. At thermodynamic equilibrium, *E*_F_ tells the occupation of the energy levels, but thermodynamic equilibrium can be perturbed by external bias or irradiation due to injection or photogeneration of holes and electrons. Therefore, the quasi-Fermi levels define the non-equilibrium densities of electrons in conduction band and holes in valence band. Generally, the quasi-Fermi level for the majority carriers approximates the equilibrium *E*_F_ because of the negligible increase of density of majority carriers. However, the quasi-Fermi level can be shifted because of the small minority carriers at equilibrium.^150^

**Fig. 10:**
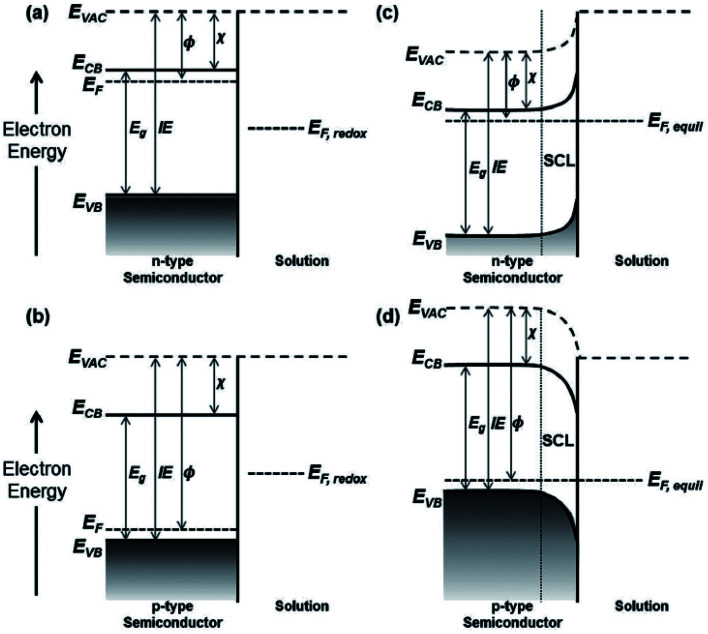
Energy levels of the semiconductor/electrolyte interface before (a and b) and after contact (c and d).^150^

When a semiconductor is in contact with another phase, the ionic interactions at the interface of the two phases cause an electrostatic adjustment in the material. To attain the equilibrium, especially at the semiconductor/electrolyte interface, the electrons flow from the phase of more negative *E*_F_ to the other, in which the semiconductor *E*_F_ matches the electrolyte *E*_F_,_redox_. This forms a space charge layer (SCL) in the semiconductor phase, which is associated with the upward bending of the band edges in the n-type semiconductor (Fig. 10c) and downward band bending in the p-type semiconductor (Fig. 10d). The SCL contributes to an internal electric field in the semiconductor, where majority carriers are forced away from the interface of semiconductor/electrolyte. Such an SCL accounts for one of the three distinct double layers, in addition to the Helmholtz layer and Gouy–Chapman layer, which are commonly present at the interface of semiconductor/electrolyte.^150^

#### Recyclability of perovskite photocatalyst

Reusability is crucial for a photocatalyst, reflecting its superiority in the photocatalysis domain. It is worth mentioning that commonly available or used photocatalysts are homogeneous photocatalysts that cannot be recycled in the developed methods. Moreover, their practical applications are limited because of complex synthesis approaches, the high cost of noble metals, conditions of air-free reaction, and modest activity. Therefore, it is essential to develop an easily produced heterogeneous photocatalyst that also exhibits the property of easy catalyst separation along with recyclability. The researchers have recently started reporting heterogeneous perovskite photocatalyst in photocatalytic organic synthesis. The perovskites-catalyzed selective oxidation of benzyl alcohols to aldehydes under visible light irradiation has been reported by a group of researchers. Another group reported the photoredox CsPbBr_3_-catalyzed oxidative coupling of thiols to disulfides and cross-dehydrogenative coupling of dialkyl H-phosphonates with tertiary amines to α-phosphoryl tertiary amines. Yan and their fellows also reported the α-acylmethylation of aldehydes starting from aldehydes and α-bromo ketones catalyzed by CsPbBr_3_ under light. The same group also used CsPbBr_3_ photocatalyst for the formation of C–C bond *via* C–H activation, formation of C–N, and C–O *via* N-heterocyclization, and arylesterification. A group of researchers prepared a heterogenous perovskite photocatalyst and used it at least five times without noticeable degradation in its activity.^151,152^

### Applications of photocatalysis

There are numerous photocatalysis applications, but during the last couple of decades, a lot of focus has been given to applications having environmental applications such as wastewater treatment, nitrogen fixation, CO_2_ reduction, air purification, and water splitting. All of these applications except air purification have been discussed in the current article.

### Degradation of water pollutants

Rapid industrial growth, mainly the textile industry, produces increased chemical wastes in the water. Industrial chemical wastes or pollutants are known as dyes. The dyes produced by the textile industry can impede plant growth, impair photosynthesis, and its accumulation in soil disturbs the food chain. The textile dyes in water also cause genetic problems and cancer. The dyes in water aggravate the regulatory functions of the different glands in humans and can also have adverse effects, like infertility and immune suppression in humans. The traditional ways to clean the dyes in water give rise to massive sludge because of adsorbent usage, which can further cause bacterial infections and skin problems. The photocatalytic degradation of the containments in the water is more reliable than the traditional methods. Recycling the adsorbents in conventional methods generally generates secondary pollutants, and recycling adsorbents also requires harsh conditions.^153–156^

The use of the photocatalyst is an eco-friendly way to degrade these water pollutants. The photocatalytic techniques utilize the advanced oxidation process (AOP) for degradation. The interaction of the light with the photocatalyst generates electron–hole pair. Generally, oxygen is employed as an electron scavenger to increase the recombination time for enhanced photocatalytic activity. The redox reaction of the photogenerated electron–hole pair gives reactive species such as (OH*, OH^−^, H^+^), and these reactive species then attack the organic pollutants and degrade them into low molecular weight products (environmentally friendly products) within no time.^153,157,158^

The heterogeneous design and the self-cleaning surface of the catalyst make recyclability much easier. The reactions occur at the catalyst's surface, followed by the desorption of the materials; thus, this method is known as a sustainable solution to address the water contamination crisis.^159,160^

**Fig. 11:**
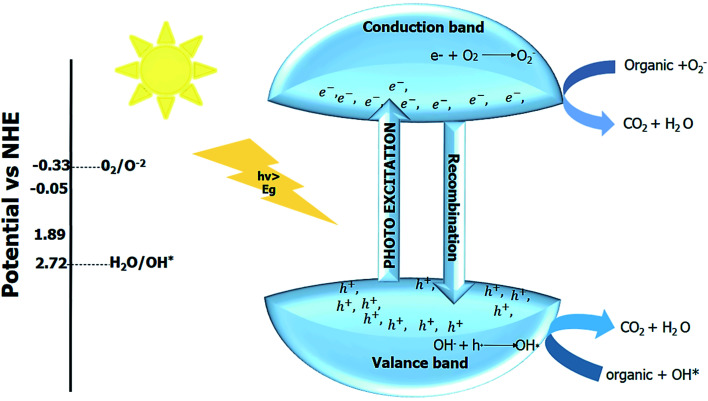
Photocatalytic pollutant degradation.

The photocatalytic activity can not only be monitored by investigating the absorption spectra of the dye molecule but also involves the investigation of the mineralized products formed due to the cleavage of the chromosphere. The mineralized products can be investigated by measuring the chemical oxygen demand (COD) and total organic carbon (TOC). The photocatalytic reaction for degradation of the containments occurs in an aqueous environment. The irradiation of the light on the aqueous system (consisting of photocatalyst + polluted water) generates photogenerated electron–hole pair in water. The oxygen or different impurities are added as an electron scavenger to increase the recombination time.^25,104,161,162^ The photogenerated electron–hole pair then migrates towards the surface of the photocatalyst and induces the reactive ions (such as OH*, O^2−^*, *etc.*). The redox potential of the created reactive ion species depends upon the bandgap of the photocatalyst. The reactive ion species then degrade the contaminant into the smaller fragments, which are then changed into green compounds. The degradation of the contaminants takes place at the surface of the catalyst. Initially, the dye gets adsorbed at the catalyst surface, and after the degradation product gets desorbed, the new pollutant is adsorbed, and this process continues until no contaminant is left. The overall reaction mechanism is shown in (Fig. 11).^30,76,135,163,164^

Many perovskite materials are used as a photocatalyst, but titanium, bismuth, and ferrite-based single and double perovskite are widely reported due to their excellent photocatalytic properties.^165^ The first reported perovskite material CaTiO_3_ exhibited photocatalytic activity and degraded pollutants. The wide bandgap of CaTiO_3_ (3.0–3.5 eV) responds only to the UV light and efficiently degrade the brilliant green (BG), methyl blue (MG), and rhodamine B (RHb).^88,166^ The synthesis route and the morphology of the CaTiO_3_ structure change the bandgap and thus affect the photocatalytic performance for pollutant degradation. CaTiO_3_ nanocuboid showed the excellent photodegradation of the RhB dye upon visible light irradiation. Zirconium (Zr) doping at the Ti site of CaTiO_3_ generates oxygen vacancies, producing defects and increasing photocatalytic activity by changing the lattice structure.^167,168^ The reported photocatalytic activity of the Zr doped CaTiO_3_ is thirteen times greater than the un-doped CaTiO_3_. Fe-doped CaTiO_3_ efficiently degraded the methyl blue using the visible light source.^166^ The doping of the Fe in the CaTiO_3_ increased the absorption ability of CaTiO_3_. The photocatalytic activity also depends upon the calcination temperature and the irradiation time of the light. Fe-doped CaTiO_3_ showed 100% photodegradation of the methyl blue at optimum condition (temperature, irradiation time, and light source).^169^ BaTiO_3_, SrTiO_3_, and other titanium-based perovskite materials have also shown better photodegradation of the pollutants.^101,102,170^

Ferrites-based perovskite (AFeO_3_, where A can be La, Bi, Ca, Sr, Gd, *etc.*) attracted researchers due to their low cost and small bandgap compared to the titanium-based perovskite.^171^ The widely used ferrite-based perovskite is bismuth ferrite (BiFeO_3_). The small bandgap (2.0–2.77 eV), excellent stability, and strong photoabsorption of BiFeO_3_ allow the efficient photodegradation of organic dyes from textile and pharmaceutical industries under visible light.^172–174^ The pure BiFeO_3_ in the sunlight showed 69% photodegradation of methyl blue. The doping at different sites of BiFeO_3_ has shown better results. Sc doped BiFeO_3_ showed 100% photodegradation of the methyl blue in 3 hours under sunlight. The improved efficiency was attributed to improved ferroelectric properties due to lattice distortion produced by doping.^98^ Mesh of BiFeO_3_ showed 98% photodegradation of the methyl blue within 4 hours. The excellent photocatalytic activity of BiFeO_3_ was due to the high interaction of the dye molecules and photocatalyst. Bi_0.90_La_0.10_Fe_0.95_Mn_0.05_O_3_ showed 97% photodegradation of Congo-red within 2 hours under the sunlight.^25^

Tantalite-based perovskite is also reported for the degradation of water pollutants. Due to excellent photochemical stability, sodium tantalite (NaTaO_3_) showed photocatalytic degradation properties. The efficiency of the NaTaO_3_ based photocatalytic system is quite less due to the wide bandgap of the material. Still, the doping of nonmetals increases its efficiency by creating a local state between the conduction and valence band. The reported photodegradation of methyl blue is 95.21% by nitrogen-based NaTaO_3_ photocatalyst using sunlight as an irradiation source.^175,176^ Different perovskite materials used for photocatalytic pollutant degradation are given in Table 2.

**Table 2:** Reported perovskite materials for photocatalytic pollutant degradation

Material	Morphology	Bandgap (eV)	Pollutants/dyes	Light source	Degradation rate
CaTiO_3_ (ref. 177)	Bare	∼3	MO	UV light	54% after 60 minutes
CaTiO_3_–graphene^178^	Composites	∼3	MO	UV light	98% after 60 minutes
C doped SrTiO_3_ (ref. 179)	Cubic particle, nanorod, nanotube	Less than 3.2	MB, MO, RhB, phenol, and BPA	Visible light	95% of MB, MO, RhB, and 70% of phenol and BPA after 3 hours irradiation
S–SrTiO_3_ (ref. 180)	Powder	<3.2	2-Propanol	500 W-Xenon lamp	After 60 min of irradiation, 80% of propanol is converted into acetone
Cu doped SrTiO_3_ (ref. 181)	Nanoparticle	2.96	Methyl blue	Visible light	66% within 120 minutes
Fe doped SrTiO_3_ (ref. 182)	—	2.6	Tetracyclin TC	Visible light	71.6% in 80 minutes
Mn-doped SrTiO_3_ (ref. 183)	Nanocubes	2.76	Tetracyclin TC	Visible light	66.7% in 60 minutes
N doped NaTaO_3_ (ref. 184)	Cubic	2.48	MB, MO	UV-visible light	95.1% in 60 minutes
Ag/AgGaO_2_ (ref. 185)	Composite		MB	Visible light	95% in 180 minutes
LaFeO_3_ (ref. 186)	Nanoparticle	2.36	MB	Visible light	100% after 60 minutes
Z-scheme MoS_2_/CaTiO_3_ (ref. 187)	Nanospheres	3.23	TC	Simulated solar light	70% in 60 minutes
p–n type (30–60% Ag_3_PO_4_)/NaTaO_3_ (ref. 188)	Crystalline	2.32–3.78	RhB	Visible light	87% in 25 minutes
BiOI/KTaO_3_ p–n heterostructure^189^	Composite	1.76–2.23	RhB and phenol	Visible light	91% after three cycles
BiFeO_3_/BiVO_4_ (ref. 190)	Nanocomposites	2.23	RhB	Visible light	69% within 120 minutes
In_2_S_3_/NaTaO_3_ (ref. 191)	Composite	2.1–4.0	TC	Stimulated solar irradiation	53.2% for 20 wt% In_2_S_3_/NaTaO_3_ within 180 minutes
(10 wt%) LaFeO_3_/SnS_2_ (ref. 192)	Composite Z-scheme heterojunction	2.11	TC	Visible light	28.8% in 120 minutes
(1.7 wt%) Ag–KNbO_3_ (ref. 193)	Nanowires	2.2–3.35	RhB	UV-visible	95% with UV in 90 minutes and 65% with VIS in 120 minutes
7% Ni-doped BiFeO_3_ (ref. 194)	Nanoparticle	∼2.28	MB	Visible light	92% within 60 minutes
LaNiO_3_ (ref. 195)	—	2.26	MO	Visible light	74.5% after 5 hours
(5 wt%)NiS/LaFeO_3_ (ref. 196)	LFO nanoparticle NiS nanosheets (heterostructure)	1.2–2.0	MO	Simulated sunlight	90.9% higher than pure LFO
NaTaO_3_/rGO (1.5%)^197^	Composite	3.87	MB	8 W UV lamp	95% after 90 minutes
N-doped NaTaO_3_ (ref. 176)	Cubic	Less than 3.94	MO	Visible light	95.21% after 14 hours
(50% wt) BiFeO_3_/V_2_O_5_ (ref. 198)	Nanoplates	2.05–2.19	MB	Visible light	96% after 120 minutes
BiFeO_3_/25% wt ZnFe_2_O_4_ (ref. 199)	Nanocomposites	2.2–1.96	MB	Visible light	96% after 30 minutes
Sm and Mn doped BiFeO_3_	Nanoparticles	1.45–2.08	MB, MV	Visible light	65%,64% after 2 hours
Carbon dots/BaZrO_3_ (ref. 200)	Hybrid nano nanomaterial	4.8	MB	UV light	90% after 60 minutes
Z-scheme LaCoO_3_/g-C_3_N_4_-60 wt% (ref. 135)	Composites	2.46	Phenol	Visible light	85% in 5 hours
CuS/Bi_2_WO_6_ (ref. 201)	Composites	1.76–2.69	RhB	Visible light	90.0% in 50 min
Bi_2_WO_6_ (ref. 202)	—	2.7–2.85	EBT	Visible light	74% in 180 min
Sm-doped Bi_2_WO_6_ (ref. 203)	—	2.4–2.5	RhB	Visible light	98.4% in 30 min
(0.3 : 1) Bi_2_WO_6_/ZnO^204^	Flower-like composite	2.6–3.2	MB, TC	Visible light	98.4% for MB in 120 min, 90% for TC in 120 min
Bi_2_MoO_6_ (ref. 205)	Nano sheets	2.6–2.9	MB	Visible light	90% of MB in 120 min
BiFeO_3_/Bi_2_Fe_4_O_9_ (ref. 206)	Nanofibers	1.96–2.15	RhB	Visible light	65% in 1.5 h
2% Ag/Bi_2_WO_6_ (ref. 207)	3D hierarchical hybrid material	—	RhB, TC	Visible light	100% in 50 min/90% in 70 min
CQD/Bi_2_WO_6_ (ref. 208)	Composite	2.6	MO, BPA	Visible/IR light	(94.1%/18.3%) in 120/90 min, (99.5%/25.5%) in 60/90
g-C_3_N_4_/Bi_2_WO_6_ (ref. 209)	Nanosheets	2.69	Ibuprofen	Visible light	96.1% in 60 min
Bi_2_WO_6_/RGO^210^	Microsphere	2.3–2.69	Phenol, MO, RhB, SMM, SN	Sunlight	65.5% in 480 min, 78.5% in 480 min, 99.5% in 480 min, 70.9% in 480 min, 57.6% in 480 min
La_2_NiO_4_/ZnO^211^	Heterosystem	1.87–3.1	MO	Sunlight	99.9% in 60 min
SnSe/LaNdZr_2_O_7_ (ref. 212)	Composites	1.69–3.34	Foron blue	Visible light	86.3% in 60 min
m-Bi_2_O_4_/Bi_2_O_2_CO_3_ (ref. 213)	Composite	1.53–2.0	RhB	Visible light	95.3% in 50 min

#### Photocatalytic water splitting

Hydrogen is considered a green and clean fuel and one of the best alternatives to fossil and other non-renewable energy resources. The photocatalytic splitting of the water produces H_2_ and O_2_ oxygen by the four-electron process. The overall reaction of water splitting is depicted in eqn (9).

9 2H_2_O → 2H_2_ + O_2_ Δ*G*° = +237 kJ mol^−1^

The positive Gibbs free energy shows that with the help of some external stimuli, the reaction proceeds in the forward direction. The use of some sacrificial layer (or agent) in combination with the photocatalyst material produces hydrogen and oxygen, and the reaction is known as hydrogen or oxygen evolution reaction. The process of producing hydrogen and oxygen from water by using semiconductor photocatalyst material and light as an irradiation source to innate the reaction is known as photocatalytic water splitting.^214–216^ The photocatalytic reaction mainly depends upon the reduction potential required for water splitting. The minimum potential energy needed to convert H_2_ and O_2_ from water is 1.23 eV.^217^

**Fig. 12:**
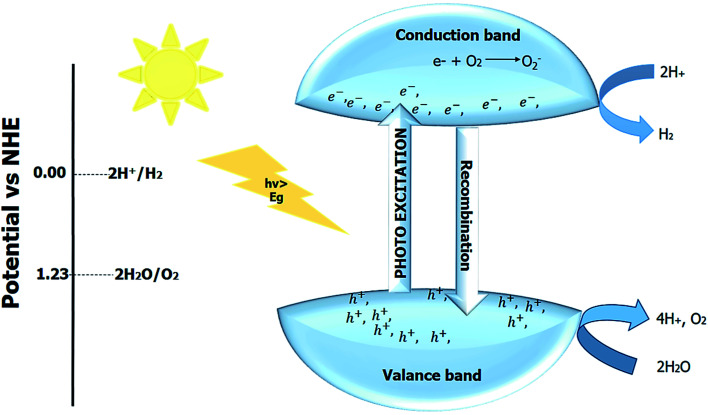
Photocatalytic water splitting.

One-step photo reaction requires only one semiconductor photocatalyst material, and the overall process is depicted in Fig. 12. The use of two photocatalytic materials to form composite or heterojunction can enhance the performance of the perovskite material. Z-scheme mainly employs two materials in photocatalytic water splitting. Z-scheme is an eight electrons process. The selection of the photocatalyst depends upon the target product. In Z-scheme, the hydrogen-evolving or oxygen-evolving photocatalysts are combined, and both catalysts perform their function separately. The mediators (solid or aqueous) help transfer charge in the Z-scheme. Direct Z-scheme is a process without any mediator. The thermodynamic requirement for the water splitting can be decreased in the Z-scheme by allowing only hydrogen and oxygen-evolving semi-conductors. Still, the kinetics of the Z-scheme reactions are challenging, and nearly half of the hydrogen and oxygen are produced compared to a one-step reaction.^218–221^

The performance of the photocatalyst can be evaluated by the quantity of H_2_ evolved and the recyclability of the photocatalyst. The reaction rate is not always proportional to the use of photocatalyst mass; therefore, the preferred way is H_2_ evolved per unit time (μmol h^−1^) with the mass utilized during reaction rather than writing the amount of H_2_ produced in a mass normalized from (μmol h^−1^ g^−1^).^222,223^ The intensity of the incident light for the photocatalytic performance is the number of photons collected per particle is directly related to the particle size. The reaction rate for the photocatalyst is proportional to the intensity of the incident light. In some cases, the rate of reaction increases with excitation intensity. In contrast, the increase in intensity in some reactions leads to decreased photocatalytic efficiency due to the second-order recombination of the photo-induced charges.^224^ At low intensity, the concentration of the photogenerated charge carrier is insignificant compared to the concentration of intrinsic majority carriers. Thus, it can also be assumed that the concentration of minority charges varies with incident light's intensity, whereas the concentration of majority charges stays nearly constant.^225–227^

The recombination of the photogenerated charges can be determined by the quasi-first-order reaction with respect to the concentration of minority carriers induced by the light source. In simple words, the order of a photocatalytic reaction at a specific intensity gives an idea about the photoexcited charge carrier concentration. For comparison, a standard parameter of the apparent quantum efficiency (AQE) and the solar to hydrogen conversion (STH) are reported for measurements of photocatalytic activity.^228–230^
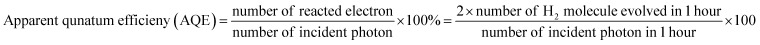

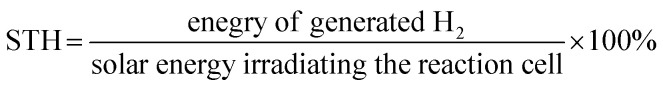


Moreover, the stability of photocatalyst for real-world applications is vital as these materials need a life cycle of about ten years with an efficiency of about 5–10% STH to meet the goal price of $ 2.00–4.00 per kg. The absorption wavelength for water splitting should be around 526 nm (2.36 eV) because below this wavelength, the photon's energy is not sufficient to achieve STH efficiency of 5–10%, considering that the reaction takes place for a lower wavelength at the AQE = 1. To meet the industrial demand, the practical need for the evolution of H_2_ under sunlight, the photocatalyst with the bandgap of less than 2.36 must be developed. The perovskites-based photocatalytic materials gained significant attention for the practical application of water splitting under UV light and visible light.^231–234^

Titanium-based perovskite materials are widely reported for photocatalytic water splitting. Doping and co-doping change the morphology and bandgap of SrTiO_3_, thus are making it a suitable visible light photocatalyst for water splitting reaction. The use of the Pt as a co-catalyst induces the Schottky barrier and can make the photocatalyst responsive to visible light. The presence of the Pt also provides the site for the proton reduction. The quantum yield efficiency by using the visible light as an irradiation source is 0.9% while using the UV light, the quantum yield efficiency of the system is 1.9%. The use of the other metals as Au, Ag, Fe, Ce, and Ni are also reported.^235–239^

Alkali tantalates are the best perovskite material reported for photocatalytic water splitting when the parameters like the doping of the metal cations and morphology (nanocubes) of tantalates-based material are optimized well. The reported hydrogen evolution by KTaO_3_ nanocubes is 375 μmol g^−1^ h^−1^. The Ag doping at the surface of the KTaO_3_ increases the hydrogen evolution in UV light. The irradiation of the UV light generates oscillations in the conduction band of absorbed silver and thus allows the easy excitation of the particles to the conduction band of KTaO_3_. The local electric field of KTaO_3_ was enhanced with the doping of the silver nanoparticles. The enhanced field promotes the electron–hole pair generation, and therefore, increases the photocatalytic splitting. The reported evolution of the hydrogen in the presence of Ag as a dopant in KTaO_3_ is 2072 μmol g^−1^ h^−1^.^240–243^ The lanthanum and ferrite-based perovskite material are also reported.^244^Table 3 lists various perovskite materials used for water splitting.

**Table 3:** Perovskite materials for photocatalytic water splitting

Material	Co-catalyst	Morphology	Amount of H_2_ and O_2_ evolved/AQE value	Bandgap (eV)	Reaction conditions	Light source
CaTiO_3_–MoS_2_-RGO^245^	None	Nanocomposite	808.0 μmol g^−1^ h^−1^ 5.4% at 365 nm	3.42–3.60	25 vol% lactic acid	Sunlight
Defected CaTiO_3_ (ref. 167	None	Nanosheets	2.29 mmol g^−1^ h^−1^	2.85	For the synthesis of the defected sheet, the hydrogenation treatment for 5 hours and 50% methanol	300 W Xe lamp
Cu-doped CaTiO_3_ (ref. 246)	None	Powder	295.0 μmol g^−1^ h^−1^	3.40–3.9	20 vol% methanol	300 W Xe lamp (*λ* > 415 nm)
Er-doped CaTiO_3_ (ref. 247)	Pt	Nanocrystal	461.25 μmol h^−1^	3.30	20 vol% methanol	300 W Xe lamp (320–390 nm)
CaTiO_3_/Pr^3+^–Y_2_SiO_5_/RGO^248^	Pt	Composite	0.19 μmol g^−1^ h^−1^/0.003% at 400 nm	—	None	300 W Xe lamp (*λ* > 400 nm)
CdSe/CaTiO^249^	Pt	Nanocomposite	3.01 mmol g^−1^ h^−1^	1.6–3.27	Na_2_S and Na_2_SO_3_	300 W Xe lamp
CaTiO_3_ (ref. 250)	Pt	—	0.39 μmol min^−1^	3.4	None	300 W Xe lamp
AgCl/Ag/CaTiO_3_ (ref. 133)	None	Nano sheets	226.53 μmol g^−1^ h^−1^	—	10 vol% methanol	300 W Xe lamp
SrTiO_3_ (ref. 251)	Pt cluster	Powder	23.0 μmol h^−1^/8.0% at 350 nm	3.2	None	300 W Xe lamp
SrTiO_3_ : C, N^252^	Pt	Nanocuboid	68.0 μmol h^−1^	2.97	20 vol% methanol	300 W Xe lamp
Cr, Ta codoped SrTiO_3_ (ref. 253)	Pt	—	122.6 μmol h^−1^/2.6% at 420 nm	2.3	10 vol% methanol	300 W Xe lamp (*λ* > 420 nm)
Pt@CdS/3DOM-SrTiO^134^	Pt	Composite	57.9 mmol g^−1^ h^−1^	2.4–3.2	10 vol% lactic acid	300 W Xe lamp
CdS/Au/3DOM-SrTiO_3_ (ref. 254)	None	Composite	5.46 mmol g^−1^ h^−1^/42.2% at 420 nm	2.4–3.2	0.1 M Na_2_SO_3_ and Na_2_S	300 W Xe lamp (*λ* > 420 nm)
CdSe/BaTiO_3_ (ref. 255)	None	Nanocube composite	53.4 μmol g^−1^ h^−1^	1.8–3.2	0.05 M Na_2_SO_3_ and Na_2_S	300 W Xe lamp (*λ* > 420 nm)
CdS/NiTiO_3_/CoS^256^	None	Nanocomposite	476.20 μmol h^−1^	2.1–2.4	Lactic acid	Vis-NIR
TiO_2_/MgTiO_3_/C^257^	Pt	Nanocomposite	33.30 mmol g^−1^ h^−1^/1.46 mmol g^−1^ h^−1^	—	30 vol% methanol	Solar light/visible light
NaTaO_3_ (ref. 258)	RuO_2_	Powder	430.0 μmol g^−1^ h^−1^	3.92–4.00	None	400 W Hg lamp
NaTaO_3_ microspheres microcubes^87^	NiO	Microsphere	0.26 μmol h^−1^/0.05 μmol h^−1^	—	None	8 W UV lamp, 254 nm
NaTaO_3_/RGO^259^	None	Composite	267.50 μmol g^−1^ h^−1^	—	0.05 M Na_2_SO_3_ and Na_2_S	250 W Hg lamp
Ag–NaTaO_3_ (ref. 260)	None	Nanocubes	3.54 μmol g^−1^ h^−1^	3.0–4.7	25 vol% methanol	300 W Xe lamp
C-doped KTaO_3_ (ref. 261)	Pt	Nanocubes	592.0 μmol g^−1^ h^−1^	—	20 vol% methanol	300 W Xe lamp
Ag–KTaO_3_ (ref. 260)	None	—	185.60 μmol g^−1^ h^−1^	2.9	25 vol% methanol	300 W Xe lamp
Porphyrin-KTa(Zr)O_3_ (ref. 262)	None		53.7 μmol h^−1^/29.4 μmol h^−1^		None	300 W Xe lamp
LiTaO_3_ (ref. 263)	None	Nanoparticles	712.0 μmol h^−1^	4.6–4.7	None	250 W high-pressure Hg lamp
Nb-substituted AgTaO_3_ (ref. 264)	Pt and Co–Pt	—	1.68 μmol h^−1^	—	None	Xe lamp
N-rGO/N–NaNbO_3_ (ref. 265)	Pt	Nanocrystal	2.34 mmol g^−1^ h^−1^/5.1% at 320 nm	3.4–3.7	20 vol% methanol	300 W Xe lamp
NaNbO_3_ wires^91^	Pt	—	26.6 μmol h^−1^	—	20 vol% methanol	300 W Xe arc lamp
C-doped KNbO_3_ (ref. 266)	Pt	—	211.0 μmol g^−1^ h^−1^	3.06	20 vol% methanol	300 W Xe lamp
CdS/Ni/KNbO_3_ (ref. 267)	None	Nanocomposite	23.5 μmol h^−1^	—	50 vol% methanol	500 W lamp
MoS_2_/C-doped KNbO_3_ (ref. 97)	Pt	—	1.30 mmol g^−1^ h^−1^	—	20 vol% methanol	300 W Xe lamp
g-C_3_N_4_/SrTiO_3_ (ref. 79)	Pt	Nanocomposite	966.80 μmol g^−1^ h^−1^	2.68–3.16	10 vol% TEOA	300 W Xe lamp (*λ* > 420 nm)
KNbO_3_/g-C_3_N_4_ (ref. 268)	Pt	Composite	180.0 μmol g^−1^ h^−1^	2.7–3.06	20 vol% methanol	300 W Xe lamp (*λ* > 420 nm)
LaFeO_3_/g-C_3_N_4_ (ref. 269)	NiS	Composite	121.0 μmol g^−1^ h^−1^/2.01% at 420 nm	2.0–2.6	10 vol% TEOA	300 W Xe lamp (*λ* > 400 nm)
BiFeO_3_/Bi_2_Fe_4_O_9_ (12.3% Bi_2_Fe_4_O_9_)^206^	None	Heterostructure nanofiber	800 μmol g^−1^ H_2_	1.96–2.15	A sacrificial layer of triethanolamine	—
Sr_2_CuWO^270^	1 wt% Pt for H_2_ production (water reduction) and 1 wt% CoO for O_2_ production (water oxidation)	Nanopowder	No H_2_ was produced, and the quantum efficiency of O_2_ produced was 0.034	2.07	A sacrificial agent such as sodium sulfite for H_2_ production	Visible light (*λ* ≥ 420 nm
g-C_3_N_4_/Ba_5_Ta_4_O_15_ (33.47 wt% g-C_3_N_4_)^218^	1 wt% Pt	Nanosheets wrapped by g-C_3_N_4_ foil/nanosheets heterostructure	60–70 μmol of H_2_ evolved in 5 hours	2.8–4.3	A sacrificial layer of oxalic acid	Visible light
Cs_2_AgBiBr_6_ (ref. 271)	2.5% RGO	Composite	489 μmol g^−1^ H_2_ in 10 h	2.77	H_2_ evolution in saturated HBr aqueous solution	Visible light
Ba_5_Ta_4_O_15_ (ref. 272)	Cr_2_O_3_/0.0125 wt% Rh	Nanoparticle	465 μmol h^−1^ H_2_ and 228 μmol h^−1^ O_2_/100 μmol h^−1^ H_2_	4.5	Ba_5_Ta_4_O_15_ prepared by the citrate method	Visible light
Zn_2_Ti_3_O_8_ (ref. 273)	5 wt% RuO_2_	Nanorods	4 μmol h^−1^ (0.1 gram) of H_2_ and 2 mmol h^−1^ (0.1 g) of O_2_	3.56	Before the photocatalytic activity, the solution is deaerated by evacuation	300 W Xe lamp
Ca_2_NiWO_6_ (ref. 274)	None	Nanoparticle	1.38 mmol g^−1^ h^−1^ O_2_	2.8	Ca_2_NiWO_6_ is prepared by a solid-state reaction	Visible light
W-doped Sr_2_FeNbO_6_(Sr_2_FeNb_1−*x*_ W_*x*_O_6_) (*x* = 0.01–0.09)^275^	0.2 wt% Pt	Nano particles	1.1–33 μmol h^−1^ of H_2_ depending upon *x* and 28–605 μmol h^−1^ of O_2_ depending upon *x*	2.17	The reaction is carried out in an aqueous methanol solution	Visible light
Cr–PbBi_2_Nb_2_O_9_ (ref. 276)	1 wt% of Pt	Layered perovskite system	9.4 μmol h^−1^ of H_2_ and 671 μmol h^−1^ of O_2_	2.63–2.88	—	Visible light
Bi_2_WO_6_ (ref. 277)	None	Nanoparticle	188.25 μmol g^−1^ h^−1^ of H_2_	3.1	1 : 1 of glycerol–water is used	Visible light
Cr_*x*_La_2−*x*_Ti_2_O_7_ (*x* = 0.01–0.05%)^216^	1.0 wt% Pt	Nanopowder	50–90 μmol h^−1^ of H_2_ produced	2.2	Methanol as hole scavenger	UV irradiation (*λ* > 200 nm)
Fe_*x*_La_2−*x*_Ti_2_O_7_*x* = (0.01–0.05%)^278^	1.0 wt% Pt	Nanopowder	32–45 μmol h^−1^ of H_2_ produced	2.6	Methanol as hole scavenger	UV-visible
Sr_2_NiWO_6_ (ref. 279)	1.0 wt% Pt	Nanoparticles	420 μmol g^−1^ h^−1^ of O_2_/8.6 at 420 nm	2.88	AgNO_3_ and FeNO_3_ as sacrificial layer	Visible light
La_2_Ti_2_O_7_ (ref. 280)	1.0 wt% NiO	Nanoparticles	400 μmol h^−1^ of H_2_ produced	< 3.0	Photoreduction reaction was performed in an aqueous CH_3_OH solution	UV-visible
MA_2_CuCl_2_Br_2_ (ref. 281)	1.0% loading of CuO	Powder	141 μmol of H_2_/gcal in 24 hours/144.11 μmol of O_2_ produced	—	Argon was introduced into the reactor to avoid the presence of oxygen and 1 mL of water was used as a reagent	Solar simulator
CsCa_2_Nb_3_O_10_ (ref. 282)	None/0.05 wt% Rh	Nanosheets	450–500 μmole of H_2_ in 2 hours/1700 μmole in 3 hours	3.6	Photoreduction reaction was performed in an aqueous CH_3_OH solution	UV light
KCa_2_Nb_3_O_10_ (ref. 283)	None	Nanosheets	550 μmol of H_2_ in 2 hours	3.6	CH_3_OH is used	UV light

#### Photocatalytic CO_2_ reduction

The atmospheric concentration of CO_2_ is increasing at an alarming rate due to increased human activities, deforestation, burning of fuels, and industrialization. The increased CO_2_ emission is causing global warming, and for environmental sustainability, it is essential to convert CO_2_ into valuable products. Mother Nature blessed humans with photosynthesis, in which chlorophyll act as a natural photocatalyst and, in the presence of sunlight, converts CO_2_ into water, oxygen, and food for plants. The photocatalyst also converts CO_2_ into valuable products in sunlight.^16,277,284,285^

Photocatalytic CO_2_ reduction requires more electrons than hydrogen evolution reaction. The end product from depending upon the number of available electrons. One of the limiting factors is the low water solubility of CO_2_. The complete reduction of the CO_2_ in water also produces H_2_. The Z-scheme or heterojunction process for CO_2_ reduction is reported frequently.^30,122^

**Fig. 13:**
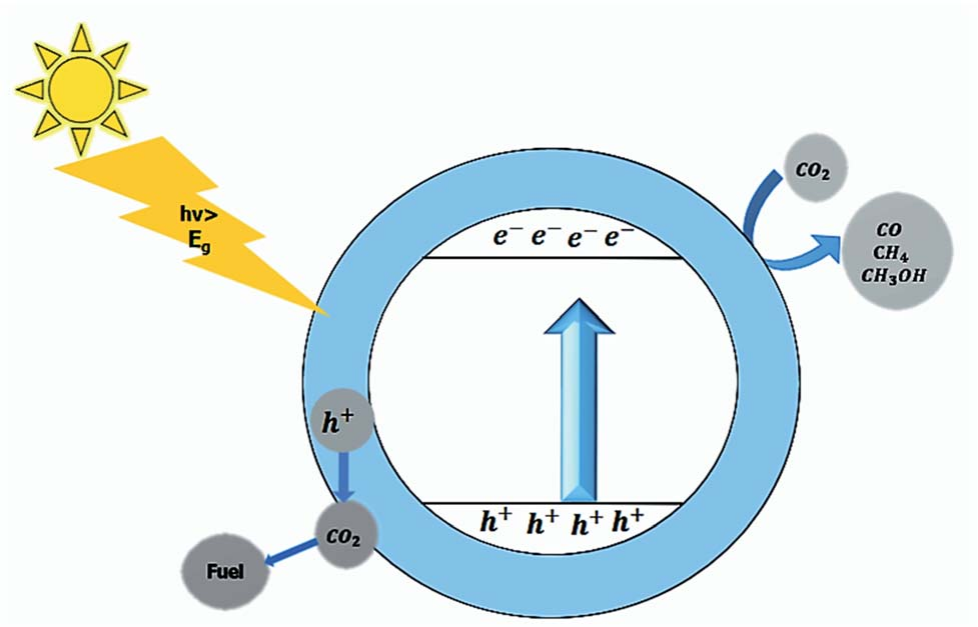
Photocatalytic CO_2_ reduction.

**Fig. 14:**
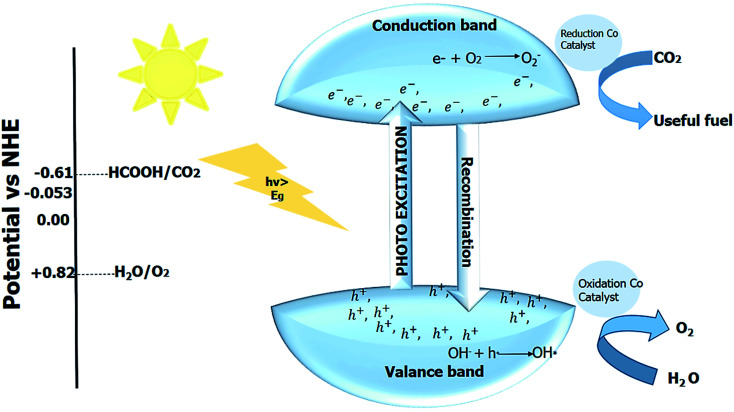
Photocatalytic reaction in presence of co-catalyst.

The first step in CO_2_ reduction involves the radical anion formation at −1.9 eV, which is impossible for most perovskite material due to less negative CB potentials of the perovskite material; therefore, high activation energy is required. The surface adsorption of CO_2_ by catalyst generates the charge CO_2_^*δ*−^ which facilities the reaction to proceed. Surface engineering can enhance the CO_2_ adsorption to the photocatalyst's surface and thus, increases the conversion efficiency. The low conversion efficiency of the CO_2_ is considered a significant concern in dealing with photocatalytic CO_2_ reduction.^286,287^ The co-catalyst can lower the activation energy resulting in enhanced photocatalytic activity. The reaction mechanism is shown in Fig. 13. In CO_2_ reduction, the CO_2_ gets adsorbed on the photocatalyst's surface, and electron–hole pair is produced upon irradiation. The photo-generated charges then move to the surface of the photocatalyst, and CO_2_ is reduced into the valuable fuel. After the reduction of CO_2_ by the photogenerated electrons and the formation of O_2_ by photogenerated holes, the reduced and the oxidized product is formed by the desorbed hole and electron, and the cycle continues. The end product depends upon the total number of electrons used in the procedure. For a better photocatalytic activity, a significant number of electrons should migrate towards the surface of the photocatalyst, and the bottom level of the photocatalyst's conduction band should be more negative than the redox potential of CO_2_. The water or additional sacrificial reagents must consume the photogenerated holes; otherwise, holes recombine with the oxygen. The photocatalytic CO_2_ reduction can be enhanced by optimizing CO_2_ adsorption, charge separation, and desorption of the products. The process of CO_2_ reduction in the presence of co-catalyst is shown in Fig. 14.^288,289^

The CO_2_ reduction by photocatalyst takes place in the presence of a water molecule, while the reaction in the gaseous or aqueous phase. The reduction potential of CO_2_ should be small compared to the reduction potential of conduction band minimum and water to obtain valuable solar fuel. During the oxidation, the oxygen molecule is formed by the reaction of the water with a valence band hole.

Calculation of the AQE is used to access the performance of the perovskite material for photocatalytic CO_2_ reduction.^290–292^

The reactions are shown as:CO_2_ + 2e^−^ + 2H^+^ → HCCOOH *E* = −0.61 VCO_2_ + 2e^−^ + 2H^+^ → CO + H_2_O *E* = −0.53 VCO_2_ + 4e^−^ + 4H^+^ → HCHO + H_2_O *E* = −0.48 VCO_2_ + 6e^−^ + 6H^+^ → CH_3_OH + H_2_O *E* = −0.38 VCO_2_ + 8e^−^ + 8H^+^ → CH_4_ + 2H_2_O *E* = −0.24 V2H^+^ + 2e^−^ → H_2_*E* = −0.41 V2H_2_O + 4h^+^ → O_2_ + 4H^+^*E* = +0.82 V

Titanium-based perovskite oxides exhibit excellent photo-stability for CO_2_ compared to the tantalates and niobates. SrTiO_3_ perovskite material is widely reported due to better charge transport properties and bandgap equivalent to TiO_2_ (3.2 eV), but the poor CO_2_ surface adsorption of photocatalyst (SrTiO_3_) creates a problem. This problem can be solved by using the metal as a co-catalyst and introducing the oxygen vacancies to improve the adsorption. In the gas phase reactor using visible light, Fe doped SrTiO_3_ photocatalyst with Pd (0.5 wt%) as a co-catalyst reduces CO_2_ to CH_4_. The yield of the product is 421, and the CO selectivity is 84%.^16,293–295^

Tantalum-based perovskites are not widely reported for CO_2_ reduction because of their wide bandgap. NaTaO_3_ photocatalyst in combination with Au (0.5 wt%) as a co-catalyst reduces CO to CH_4_ fuel in the gas phase reactor and uses UV-vis light as the irradiation source. The selectivity of CO is 96%, but the yield is small.^296^Table 4 lists perovskite materials used for CO_2_ reduction.

**Table 4:** Perovskite materials for photocatalytic CO_2_ reduction

Perovskite	Co-catalyst	Morphology	Band gap (eV)	Synthesis method/reaction condition	Product	Product concentration or conversion efficiency	Light source
NaTaO_3_ (ref. 297)	2 wt% CuO	Nanocubes	4.1	The hydrothermal method is used for catalyst synthesis. And co-catalyst is loaded *via* impregnation method/	Methanol and acetone	137.48 μmol g_cat_^−1^ h^−1^	UV-visible
						335.93 μmol g_cat_^−1^ h^−1^	
KTaO_3_ (ref. 298)	None	Nanoflakes	3.6	Perovskite material is prepared by solid-state reaction	CH_4_	19.35 ppm g_cat_^−1^ h^−1^	UV-visible
NaNbO_3_ (ref. 299)	1.5 wt% Pt	Nanoparticles (cubic)	3.29	Photocatalytic activity is carried out in a gas-phase reactor	CH_4_ and H_2_	0.486 μmol g_cat_^−1^ h^−1^	UV-visible
						127 μmol g_cat_^−1^ h^−1^	
BiFeO_3_–ZnO (ref. 300)	None	Composites	2.1–3.2	Photocatalytic activity is carried out in a gas-phase	CH_4_	The conversion efficiency of CO_2_ into CH_4_ is 21%	UV-visible
Au–SrTiO_3_ (ref. 301)	0.5 wt% Rh	Nanoparticles	—	Ru is loaded by the impregnation method, and at optimized conditions, 0.5 wt% of Au is used	CO, H_2_, and CH_4_	66.8 μmol g_cat_^−1^ h^−1^	Visible light
						50.5 μmol g_cat_^−1^ h^−1^	
						2.8 μmol g_cat_^−1^ h^−1^	
Basalt fiber@perovskite PbTiO_3_ (ref. 302)	None	Core–shell composites	1.92	The hydrothermal method is used for catalyst synthesis	CH_4_	290 μmol g^−1^ L^−1^ in 6 hours	UV light
BiFeO_3_/ZnS (ref. 303)	None	Nanocomposites	2.5	The reaction is carried out in a gas phase reactor	CO, CH_3_OH	The conversion efficiency of CO_2_ into CO and CH_3_OH is 24	UV-visible
g-C_3_N_4_/KNbO_3_ (ref. 79)	None	Composites	2.7–3.2	KNbO_3_ is synthesized by hydrothermal, and g-C_3_N_4_ powder is deposited by using the sonication method	CH_4_	1.94 μmol g^−1^ h^−1^	Visible light
N-doped LaFeO_3_ (ref. 304)	None	Nanocomposites	1.82	—	CH_4_, CO, O_2_	∼110 μmol g^−1^ h^−1^	Visible light
						150 μmol g^−1^ h^−1^	
						230 μmol g^−1^ h^−1^	
RuO_2_ on SrTiO_3_ (ref. 305)	Ru 0.1–0.4 wt%	Nanoparticles	2.7	The reaction is carried out in a gas phase	Ethanol	80 μmol g^−1^ h^−1^	Simulated sunlight
BaCeO_3_ (ref. 306)	Ag cocatalyst (0.3 wt%)	Nanoparticles	3.2	Pechini method is used to deposit the co-catalyst	CH_4_	0.55 μmol g^−1^ h^−1^	UV light
BaZrO_3_ (ref. 200)	0.5 wt% Cu	Nanoparticles	3.2	The photocatalytic reaction is carried out in a cylindrical quartz cell	CH_4_	0.98 μmol g^−1^ h^−1^	UV light
C-doped LaCoO_3_ (ref. 307)	None	—	2.16	Pechini method is used to deposit the co-catalyst	HCOOH	A minimal amount of HCOH	UV-visible
LaNi_*x*_Co_1−*x*_O (*x* = 0.4)^308^	None	Nano particles	1.42	Sol–gel combustion method is used to prepare the catalyst	CH_4_–CH_3_OH	678.57 μmol g^−1^, 20.83 μmol g^−1^ in 6 h	Visible light
H_2_SrTa_2_O_7_ (ref. 309)	0.5 wt% Ag	Layered perovskite structure	3.75	H_2_SrTa_2_O_7_ photocatalyst was prepared by PC and ion-exchange methods, and a photo deposition method was used to load Ag co-catalyst on HST	CO and H_2_	0.39 μmol g^−1^ h^−1^ of CO and 0.25 of H_2_ μmol g^−1^ h^−1^	UV light (*λ* > 200 nm)
*x*Bi_2_WO_6_/BiOI (*x* = 8%)^123^	None	Nano-composites	2.2–2.9	CO_2_ is reduced to give CH_4_ experiment is conducted into gas phase reactor	CH_4_/CO	18.32 μmol g^−1^ of CH_4_ and 320.19 μmol g^−1^ in 8 hours	Visible light
Bi_2_WO_6_ (ref. 310)	None	Nano sheets	2.7	CO is reduced to give CH_4_ experiment is conducted into gas phase reactor	CH_4_	19 ppm g^−1^ h^−1^ of CH_4_	Visible light
ALa_4_Ti_4_O_15_, A = Sr, Ca^78^	Ag	Layered perovskite structure	3.79–3.85	Catalyst is loaded *via* liquid-phase reduction and impregnation method	CO/O_2_/H_2_	10 μmol h^−1^ of H_2_ and 16 μmol h^−1^ of O_2_, 22 μmol h^−1^ of CO	A 400 W high-pressure mercury lamp, an inner irradiation quartz cell
BaLa_4_Ti_4_O_15_ (ref. 78)	0.5–2% Ag	Layered perovskite structure	3.9	Catalyst is loaded *via* liquid-phase reduction	H_2_, O_2_, CO	20–3.2 μmol h^−1^ of H_2_, 5.7–16 μmol h^−1^ of H_2_ and 5.00–22 μmol h^−1^ CO	A 400 W high-pressure mercury lamp, an inner irradiation quartz cell
Bi_2_WO_6_ (ref. 311)	0.5% wt PtO_*x*_	Ultra-thin nanosheets	—	The PtO_*x*_/Bi_2_WO_6_ was prepared by photoreduction method	CH_4_	108.8 ppm g^−1^ h^−1^	500 W Xe lamp as a light source
Bi_4_O_5_Br_2_ (ref. 312)	None	Ultra-thin nanosheets/bulk	2.64–3.05	Ultra-thin sheets are prepared by precursor method	CO	63.13 μmol g^−1^ of CO in 2 hours/27.56 μmol g^−1^ of CO in 2 hours	UV-visible light
A_3_Bi_2_I_9_ (Cs_3_Bi_2_I_9_)^284^	None	Nanocrystals	2.2	Gas-phase reaction the photoreduction to carbon take place at the gas–solid interface, the reaction medium was CO_2_ and H_2_O vapors	CH_4_/CO	14.9 μmol g^−1^ of methane and 77.6 μmol g^−1^ of CO	32 W UV lamp (*λ* = 305 nm)
Cs_2_AgBiBr_6_ (ref. 313)	None	Nanocrystals	1.72	Medium in which reaction is carried is ethyl acetate solvent	CH_4_/CO	14.1 μmol g^−1^ of methane and 9.6 μmol g^−1^ of CO	100 W Xe lamp

#### Nitrogen fixation

Nitrogen plays a vital part in building biomolecules such as amino acids, proteins, and other molecules. Earth's atmosphere has 78% of the dinitrogen (N_2_). Dinitrogen air converts into ammonia by the process known as nitrogen fixation. Plants and soil then use the ammonia formed by atmospheric nitrogen to build several processes. The mechanism to create ammonia by nitrogen is the Haber cycle.^314^ At standard conditions, the ammonia formation is a thermodynamically spontaneous process (Δ*H* = −92.2 kJ mol^−1^). Due to its high ionization energy of 15.58 eV and low electron affinity, the dinitrogen in the air is highly stable; therefore, the atmospheric dinitrogen in the normal conditions cannot be used to prepare the ammonia unless some catalyst or some industrial fixation is used. In industry, by using iron or ruthenium as a base catalyst at high temperatures, the dinitrogen is converted into ammonia by the Haber–Borsch process. But even after the century's advancement, the production of the indusial fixation is still quite low. The NH_3_ is one of the essential components in manufacturing fertilizers and hydrogen storage. Therefore, it is essential to synthesize an alternative green route to deal with energy crises and global warming.^77,315^

Recently the photocatalyst material for nitrogen fixation has gained much attention from researchers. The nitrogen fixation by the photocatalyst utilizes only sunlight and water in the presence of the dinitrogen. The six electrons do the complete conversion of N_2_ into NH_3_ due to the high activation energy of the process, which makes the process completely impractical. Furthermore, direct electron transfer and proton-coupled electron transfer are not possible for the semiconductor material. Hence, photocatalyst design and surface engineering play an important role in nitrogen fixation.^316–318^ The reaction by using the photocatalyst is designed to reduce the activation energy and weakens the N≡N bond. Light irradiation generates the electron–hole pair. These photogenerated charge carriers then move to the photocatalyst's surface, and at the conduction band, the dinitrogen is reduced to the ammonia by multi-step transfer of the electron and proton available from the water. The hole in the valence band oxidizes the water molecule and gives O_2_. The chemisorption of the N_2_ and H^+^ ions occurs on the surface of the conduction band accompanied by the association or dissociation by H_2_ incorporation of N_2_ molecules absorption and NH_3_ formation. Lastly, desorption of NH_3_ from the photocatalyst's surface after the ammonia formation occurs.^85,319–321^

The nitrogen fixation in the presence of the photocatalyst takes place in two ways, either by the associative way or by the dissociative way. The breakage of the N≡N bond occurs in the dissociative pathway before adding the hydrogen atom as a reducing agent, which is impossible in an ambient environment.^322^ In an associative pathway, breaking the N≡N bond is unnecessary, so nitrogen fixation is possible at ambient conditions. The nitrogen is adsorbed at the surface of the photocatalyst and then converted into the NH_3,_ which is then desorbed after the breakage of the N–N bond. In the associative pathway, the hydrogenation takes place with the sideway nitrogen, which is not directly attached to the surface of the photocatalyst, which generates the NH_3_.^323,324^ The remaining nitrogen attached photocatalyst's surface undergoes hydrogenation and generates NH_3_. But this route is challenging because the chemisorption of nitrogen molecules on the surface of the catalyst is not easy; therefore, surface engineering is an important parameter to enhance the photocatalyst activity for nitrogen fixation.^8,321,325^ The yield and the efficiency of the reaction are given by the apparent quantum efficiency (AQE) and can be calculated as;^326^
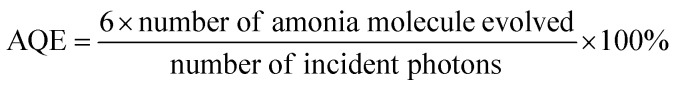


Limited numbers of the perovskite materials are reported due to the less AQE and yield. The reported materials are SrTiO_3_, KNbO_3_, LaCoO_3,_ and layered double perovskite Bi_2_WO_6_.^129,327–329^ The efficiency of the perovskite material is less due to the selectivity of N_2_ and adsorption of N_2_ at the surface of the photocatalyst material; however, more research is needed in the field of photocatalytic nitrogen fixation to compete with the available industrial method of nitrogen fixation (Haber cycle).^319,330^ Some of the reported perovskite photocatalyst materials for nitrogen fixation are listed in Table 5.

**Table 5:** Perovskite materials in photocatalytic nitrogen fixation

Material	Band gap (eV)	NH_3_ concentration/generation rate	Light source	Reaction conditions
BaTiO_3_ (ref. 331)	3.2	0.09 mg h^−1^ L^−1^	UV-visible	Water as the proton source in the process of photocatalysis
Defective La_2_TiO_5_ (R-LTO)^332^	4.07	158.13 μmol g^−1^ h^−1^	Simulated sunlight	Defects at the surface of the LTO are introduced by NaBH_4_ reduction
CeO_2_–BiFeO_3_ (ref. 320)	—	117.77 μmol g^−1^ h^−1^	UV-visible	Deionized water + nitrogen
LaCoO_3_ : Er^3+^/ATP^328^	2.88–3.45	71.51 μmol g^−1^ h^−1^	Visible	Water + nitrogen and ethanol as a sacrificial layer
Ag/KNbO_3_ (0.5% Ag)^333^	3.13	385.0 μmol g^−1^ h^−1^ L^−1^	Simulated sunlight	Ethanol as a sacrificial layer
NiS/KNbO_3_ (5% NiS)^129^	3.11	155.6 μmol g^−1^ h^−1^ L^−1^	Simulated sunlight	Ethanol is used as a hole scavenger
TiO_2_/SrTiO_3_/g-C_3_N_4_ (ref. 125)	2.75–3.1	2192 μmol g^−1^ h^−1^ L^−1^	Simulated sunlight	Methanol + nitrogen and ethanol as a sacrificial layer
CaTiO_3_ (ref. 334)	3.49	236.12 μmol g^−1^ h^−1^	Natural sunlight irradiation	3D leaf-templated defective CaTiO_3_ is prepared by using NaBH_4_ + nitrogen environment

## Double perovskite and photocatalysis

**Fig. 15:**
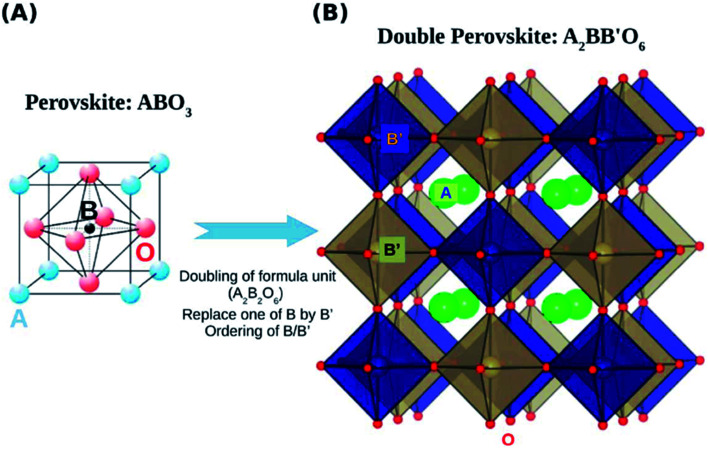
Schematic representation of a double perovskite structure (B), derived starting from a perovskite structure (A).^49^

Double perovskites can accommodate different cations at the A and B sites, forming AA′BB′O_6_ (Fig. 15). Accommodation of other cations at the A and B sites can alter the double perovskite's photophysical properties to a great extent. Among the binary oxides, only a few double perovskites exhibit visible region bandgap because of their small bandgap, such as Fe_2_O_3_, WO_6_, Bi_2_O_3_, *etc.* However, for hydrogen evolution, these materials have deficient conduction band potential. Some materials also have low mobility of the photoexcited carriers and poor stability. Many binary oxides show efficient photocatalytic activity under UV irradiation because of their wide bandgap. Complex compounds with a combination of ‘narrow bandgap’ and ‘wide bandgap’ materials can make use of properties of both types, and therefore, can be exploited as visible light photocatalysts.^124,125^

The researchers have investigated photocatalytic activity of rare earth and bismuth-based double perovskites under visible light. Double perovskites Ba_2_XBiO_6_ (X = Ce, La, Nd, Pr, Eu, Sm, Dy, Gd) were synthesized and degraded MB. Researchers reported that rare-earth cation-dependent compounds (Ba_2_SmBiO_6_, Ba_2_EuBiO_6_, and Ba_2_CeBiO_6_) showed significant photocatalytic activity. CaCu_3_Ti_4_O_12_ possessed an indirect bandgap of 1.27 eV, and Pt loaded CaCu_3_Ti_4_O_12_ showed the degradation of MO under visible irradiation. Double perovskite materials such as Sr_2_CoWO_6_, Ba_2_CoWO_6_, and Sr_2_NiWO_6_, Ba_2_NiWO_6_, are reported to be stable for O_2_ evolution with sacrificial agents.^124,125^ Although the photophysical properties of certain double perovskites also have been investigated and reported, still a lot of efforts are required before utilizing these materials on an industrial scale.

## Summary

In summary, the abundantly available sun energy can be harvested through photocatalysis to deal with the concerns pertaining to the environment and humankind. This article aimed to discuss the imperative properties of perovskite materials which play vital role in photocatalysis, and will assist in understanding the fundamentals of photocatalytic mechanisms involved in designing highly efficient photocatalysts. The three altercation sites (A, B, and O sites) of perovskite materials ma them suitable for numerous applications, particularly photocatalysis. These sites help in tailoring chemical, physical, optical, and photocatalytic properties for desired photocatalytic reactions. The structural and compositional suppleness in perovskite photocatalyst strongly affects photo-generated carriers' mobility, separation, and recombination. The current article also describes the defect engineering in perovskite materials for enhanced photocatalytic performance. Recently, defect engineering gained much attention among researchers because it resulted in visible light photocatalytic activity without doping. Furthermore, surface defects provide reactive sites beneficial for a process like nitrogen fixation at ambient conditions. The review also provides an insight into the different applications of photocatalysis, including wastewater treatment, water splitting, CO_2_ reduction, and nitrogen fixation. The critical perovskite materials for each photocatalytic application are also listed with their properties in this study. Overall, it can be inferred that although perovskite oxide-based materials have exhibited significant photocatalytic performance still, extensive challenges are ahead of their design, fabrication, cost, and efficiency for industrial and large-scale production. The biggest challenge in the industrialization of photocatalyst technology is the development of an ideal photocatalyst, which should possess four features, including high photocatalytic efficiency, a large specific surface area, full utilization of sunlight, and recyclability. Nonetheless, there is no doubt that perovskite-based oxide materials will be investigated more intensively in the coming years due to their exceptional properties and applications for a sustainable future.

## Author contributions

All authors verify their contribution to the current review article as follows: design, study conception, and supervision of the whole article is done by Muneeb Irshad and Quar tul Ain; data collection is done by; Asif Nadeem Tabish, Muhammad Usman, Masood ul Hassan Farooq, Mohammed A. Assiri, Muhammad Imran; analysis and interpretation of results by; Muneeb Irshad, Quar tul Ain, Muhammad Zaman, Muhammad Zeeshan Aslam, Naila Kousar, Muhammad Asim, Muhammad Rafique, and Khurram Siraj; draft manuscript preparation and proofreading by; Muneeb Irshad, Quar tul Ain, and Muhammad Zaman. Moreover, Mohammed A. Assiri and Muhammad Imran provided the necessary funding to complete the article. All authors reviewed the results and approved the final version of the manuscript.

## Conflicts of interest

There are no conflicts to declare.

## Supplementary Material
